# Supplementary material for: Enhancing electrochemical carbon dioxide capture with supercapacitors
Source: Nat Commun. 2024 Sep 8;15:7851. doi: 10.1038/s41467-024-52219-3 (PMC11381529; doi:10.1038/s41467-024-52219-3)
Supplement: Supplementary file 1 — Supplementary Information [file 41467_2024_52219_MOESM1_ESM.pdf]

Supplementary Information

Enhancing Electrochemical Carbon Dioxide Capture with Supercapacitors

Zhen Xu,[1] Grace Mapstone,[1] Zeke Coady,[1] Mengnan Wang,[2] Tristan L. Spreng,[1] Xinyu Liu,[1] Davide Molino,[1,3] Alexander C. Forse[1,*]

[1] Yusuf Hamied Department of Chemistry, University of Cambridge, Cambridge CB2 1EW, United Kingdom.

[2] Department of Chemical Engineering, Imperial College London, London SW7 2AZ, United Kingdom.

[3] Politecnico di Torino, Dipartimento di Scienza Applicata e Tecnologia (DISAT), Corso Duca degli Abruzzi, 24, Torino 10129, Italy.

Correspondence to: acf50@cam.ac.uk

Table of Contents:

Supplementary Methods

Supplementary Equations 1-10

Supplementary Figures 1-33

Supplementary Tables 1-3

Supplementary References

Supplementary Methods

Calculations

The specific discharge capacitance values of the working electrodes were calculated from GCD according to **Supplementary Equation (1)**, as follows:[1]

(1) $C_{electrode} = 2\frac{I\Delta t}{m\Delta U}$

where $C_{electrode}$ (F g^{-1}) refers to the specific discharge capacitance of the working electrodes, I (A) is the constant charge/discharge current, ΔU (V) is the cell voltage, m (g) is the mass of active material loaded on the working electrodes, and Δt (s) is the charge/discharge time. The cell voltage of the whole device is:

(2) $\Delta U = U_{max} - IR_{drop} - U_{min}$

Where U_{max} and U_{min} are the maximum and minimum voltage applied, respectively.

The input/output energy values of the full device were obtained using **Supplementary Equation (3)**, as follows:[2]

(3) $E_{in/out} = \frac{I}{m}\int_{t_1}^{t_2} U(t)dt + \frac{U}{m}\int_{t_3}^{t_4} I(t)dt$

where $E_{in/out}$ (kJ kg^{-1}) is the input/output energy normalized by the active mass of the working electrode. I (A) is the constant charge/discharge current, m (g) is the active mass of the working electrode, and U (V) is the constant voltage. U(t) (V) is the voltage that changes with time under galvanostatic discharging or charging, t_1 (s) is the start time of galvanostatic charge/discharge processes, and t_2 (s) is the end time. I(t) (A) is the current that changes with time during voltage hold, t_3 (s) is the start time of voltage hold, and t_4 (s) is the end time.

The specific gravimetric CO_2 adsorption capacity (C_{CO2}, $mmol_{CO2}$ kg^{-1}) was calculated by taking the difference between the maximum and minimum peaks of the gas amount (Δn, mol) in the reservoir and normalized by the active mass of the working electrode. As there is a slight irreversible decrease in the overall pressure curve from side reactions, an average of two minimum points is used to avoid the error from the irreversible pressure changes when calculating adsorption capacity. This was converted from the pressure transducer data (smoothed every 100 seconds for all experiments) using the ideal gas law:[2]

(4) $n = \frac{pV}{RT}$

(5) $\Delta n = \frac{n_{max_1} + n_{max_2}}{2} - n_{min}$

(6) $C_{CO_2} = \frac{\Delta n}{m} \times 10^6$

where p, V and T are the pressure (Pa, 10^{-5} bar), volume (m^3, 10^6 mL) and temperature (K) respectively, and n is the gas amount (mol). R is the ideal gas constant (8.31451 m^3 Pa mol^{-1} K^{-1}), and m (g) is the active mass of the working electrode. In a completed cycle of CO_2 adsorption and desorption, n_{max1} and n_{max2} represent the two maximum peaks of the gas amount, and n_{min} represents the minimum peak of the gas amount. The overall adsorption capacity was

taken as the mean of adsorption capacities for each cycle, and the error was calculated using a 95% confidence interval with the Student's t-test.

The volumetric performance is a critical metric for space-limited applications. Our current setup employs a commercial CR2032 coin cell with a fixed volume (Diameter: 20 mm, Thickness: 3.2 mm), limiting our ability to properly evaluate the volumetric performance of the whole device. The future development involving pouch cells or stacked cells with thinner polymer-based separators (*e.g.,* Celgard®), could enable more accurate investigation and improvement in the volumetric performance at the whole device level. At the current stage, the volumetric CO_2 adsorption capacity was calculated based on the gravimetric CO_2 adsorption capacity and the density of the working electrode here:

(7) $C_{CO_2-vol} = \frac{C_{CO_2}}{\rho}$

where ρ is the density (kg L^{-1}) of the working electrode. For YP80F electrodes, the density is around 0.53 kg L^{-1}.

The volume of the gas reservoir (V_1, mL) in the gas cell was calculated during the process of dosing CO_2 into the cell, based on pressure measurements of the added gas using Boyle's Law ($P_1V_1 = P_2V_2$). For each gas cell, the volume in a section of pipe between two valves was known from a prior calibration (A mL) (**Supplementary Fig. 33**). First, the gas between two valves was removed using a vacuum, and the gas amount in the main gas reservoir was proportional to P_1V_1, where P_1 (bar) was read from the pressure sensor (**Supplementary Fig. 3**g). After that, we allowed gas to enter the evacuated portion of the known volume (A mL). During this process, the total amount of gas remained the same (therefore, $P_1V_1 = P_2V_2$) but the pressure decreased to P_2 (bar) (**Supplementary Fig. 3**g). As V_2 (mL) was equal to the sum of V_1 (mL) and A (mL), the volume of the reservoir (V_1, mL) was calculated using **Supplementary Equation (8)**:

(8) $V_1 = \frac{P_2A}{P_1-P_2}$

This step was also used as the leaking test to make sure there was no leaking of the gas cell. If the gas cell leaks, the pressure will not be maintained at the low pressure of P_2 (bar) < 1 bar (**Supplementary Fig. 3**g).

The specific electrical energy consumption (E, kJ mol_{CO2}^{-1}) and adsorption rate (R_{CO2}, $mmol_{CO2}$ kg^{-1} h^{-1}) were calculated using **Supplementary Equations (9)** and **(10)**, as follows:[3,4]

(9) $E = \frac{E_{in}-E_{out}}{C_{CO_2}} \times 10^3$

(10) $R_{CO_2} = \frac{C_{CO_2}}{t_{charging}/3600}$

where $t_{charging}$ is the total charging time (including the voltage hold step).

Supplementary Figures and Tables

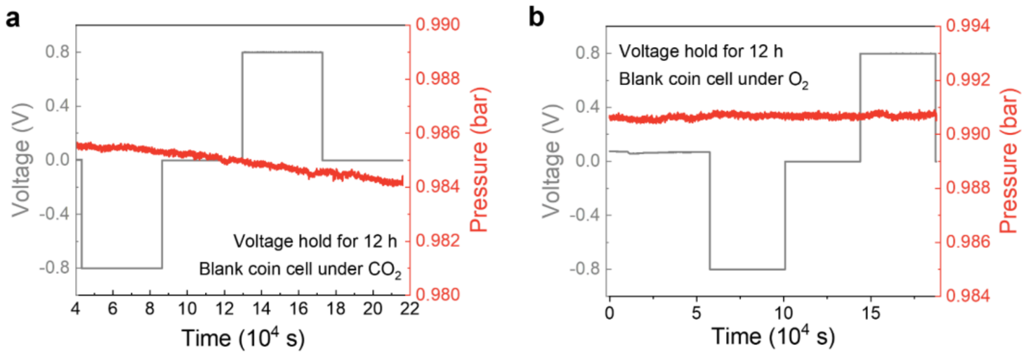

Supplementary Fig. 1. Electrochemical measurements of "blank" coin cells (without activated carbon electrodes) under CO_2 and O_2. Overall galvanostatic charge and discharge (GCD) curves (grey) and corresponding pressure curves (red) of the blank coin cell only with stainless steel spacers, spring, separator and electrolyte using 12 h voltage holds at 0 V and ±0.8 V under **(a)** pure CO_2 and **(b)** pure O_2. Notes: A control experiment with a blank coin cell was carried out to investigate the origin of the irreversible pressure drop observed under CO_2 during charging and discharging (**Supplementary Fig. 3**a). The irreversible reaction of CO_2 with the stainless-steel coin cell parts occurs with applied voltage holds as the pressure drops with a slope of –1.2 mbar per day (**Supplementary Fig. 1**a). Therefore, together with proofs shown in **Supplementary Fig. 3**f and g, the irreversible pressure decrease under CO_2 is related to the side reaction between coin cell parts, CO_2 and electrolyte rather than CO_2 equilibrium or leaking. However, in **Supplementary Fig. 1**b, no pressure increase or decrease is observed, indicating that the side reaction with O_2 occurs solely at the carbon electrode (**Supplementary Fig. 21** and **22**) and not at the stainless-steel coin cell parts or the glass fiber separator.

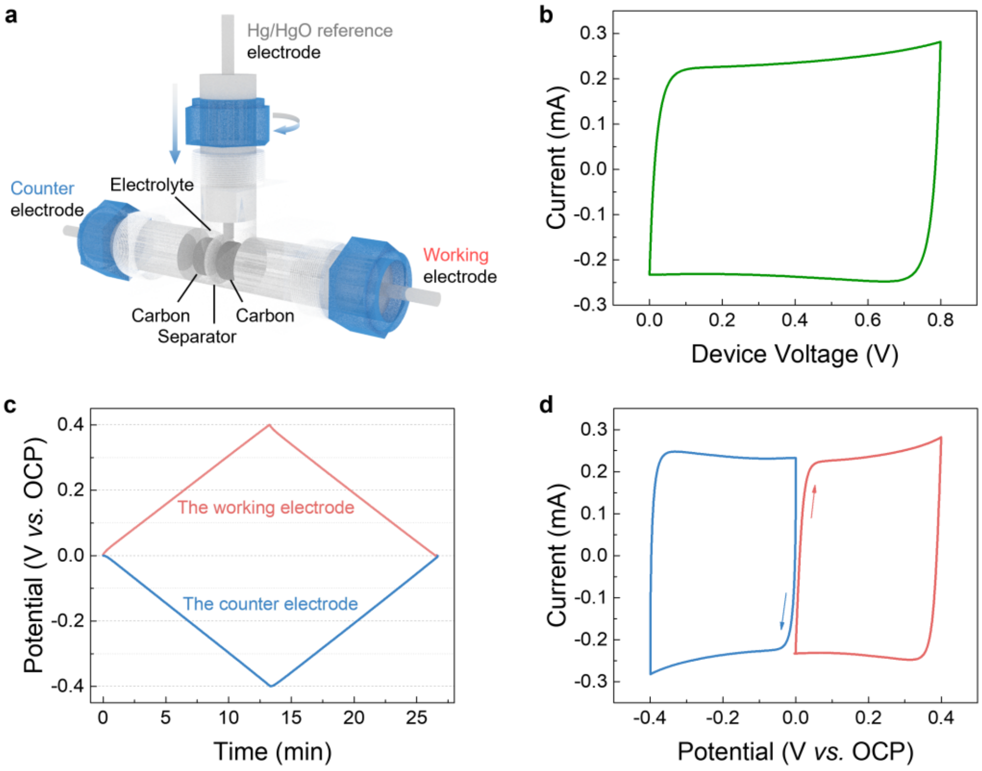

Supplementary Fig. 2. Three-electrode measurement of symmetric supercapacitors with YP80F. (a) Schematic illustration of the three-electrode Swagelok cell setup using two identical carbon working and counter electrodes together with the Hg/HgO reference electrode. **(b)** Cyclic voltammetry (CV) of the device from 0 to 0.8 V at the scan rate of 1 mV s^{-1}. The corresponding potential changes of the working electrode (red) and the counter electrode (blue) during the CV measurement *versus* **(c)** time and **(d)** current. Notes: As shown in **Supplementary Fig. 2,** the device voltage of 0.8 V is equally allocated to each electrode when we employed two identical carbons as the working and counter electrodes, respectively. However, we cannot fully rule out the effects of carbon structures or electrolyte compositions on potential distributions in other experiments. Here the open-circuit potentials (OCP) of the working electrode and the counter electrode are both 0.11 V *versus* the potential of the Hg/HgO reference electrode. To facilitate the visualization of equally allocated potentials between the working and counter electrodes, their varied potentials are plotted *versus* OCP.

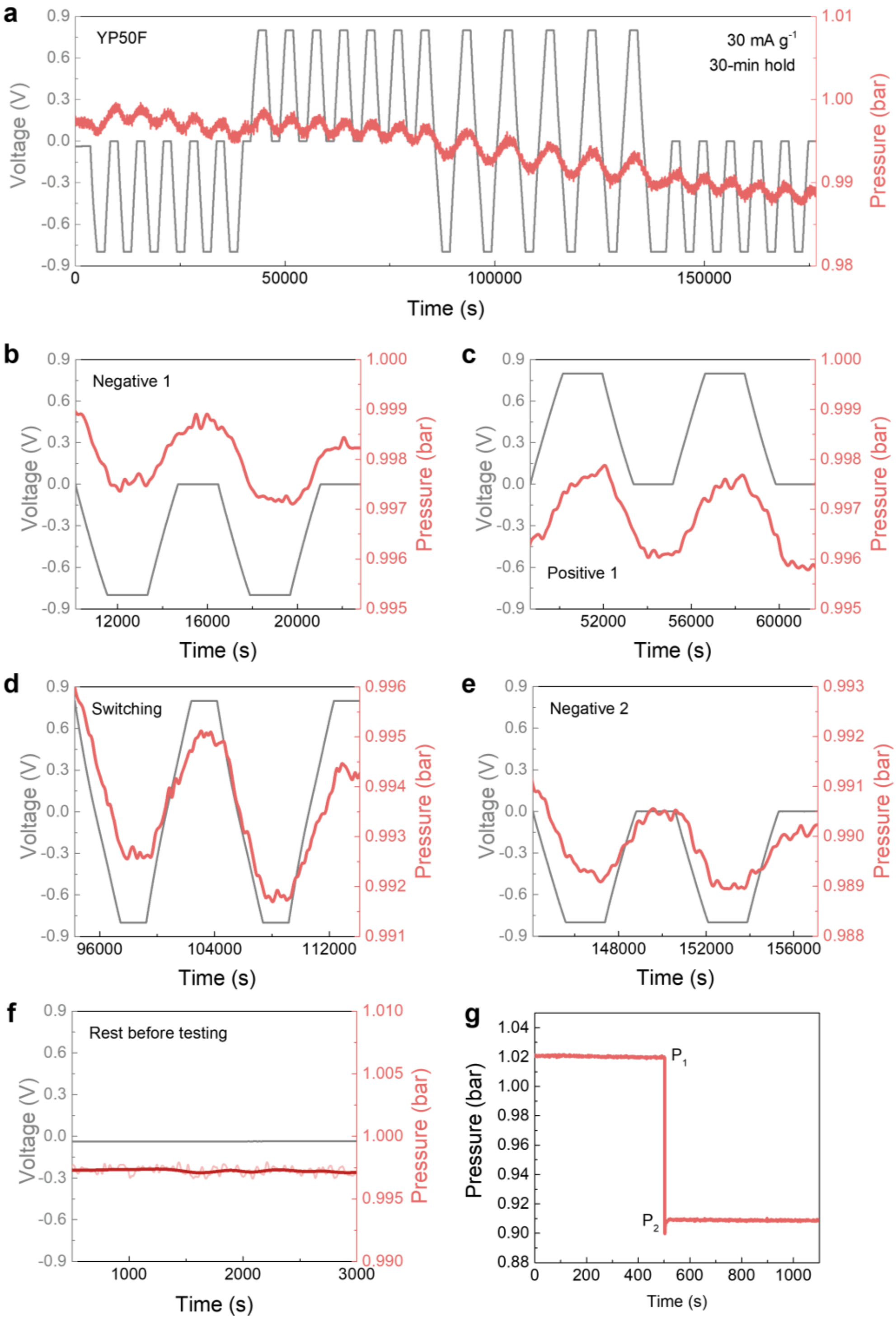

Supplementary Fig. 3. Electrochemical CO_2 capture measurement of YP50F by varying voltage under CO_2. (a) Overall GCD curves (grey) and corresponding pressure curves (red) of the device using YP50F electrodes for CO_2 sorption in negative, positive and switching charging modes (under CO_2, at the current density of 30 mA g^{-1}, with a 30-min voltage hold after the charge or discharge process). **(b-e)** Zoomed GCD curves (grey) and smoothed pressure curves (averaged every 100 sec, red) of the YP50F electrode under CO_2 at the current density of 30 mA g^{-1} in different charging modes, all with 30-min voltage holds. (Negative 1 and 2

represent the first and second negative charging parts, respectively). **(f)** Zoomed GCD curves (grey), original pressure curves (light red) and smoothed pressure curves (averaged every 100 sec, dark red) of the YP50F electrode under CO_2 during the rest before testing. **(g)** Pressure curves before and after gas was allowed to enter the evacuated portion between the two valves of the gas cell. Notes: During the 1 h rest of the gas cell, the CO_2 pressure stabilizes at a certain level below 1 bar (**Supplementary Fig. 3**f), and the system has reached an equilibrium state after precycling. During the volume calibration process (**Supplementary Fig. 3**g), P_2 was below 1 bar, and no leaking of the gas cell was observed. The pressure curve exhibits a relatively obvious downward trend during electrochemical CO_2 capture measurements with a 30-min voltage hold (**Supplementary Fig. 3**a), which results from the corrosion of stainless-steel current collectors with CO_2 and electrolyte under applied voltage and current. The long voltage hold or slow charge rate provides enough time to allow the side reactions to happen. Therefore, this downward trend in pressure curves is also observed in **Supplementary Fig. 19**a which used the current density of 1 mA g^{-1}. The potential solution to this issue is the use of a protective layer to avoid the contact between electrolyte and stainless-steel coin cell parts, and also to avoid the use of voltage holds.[5]

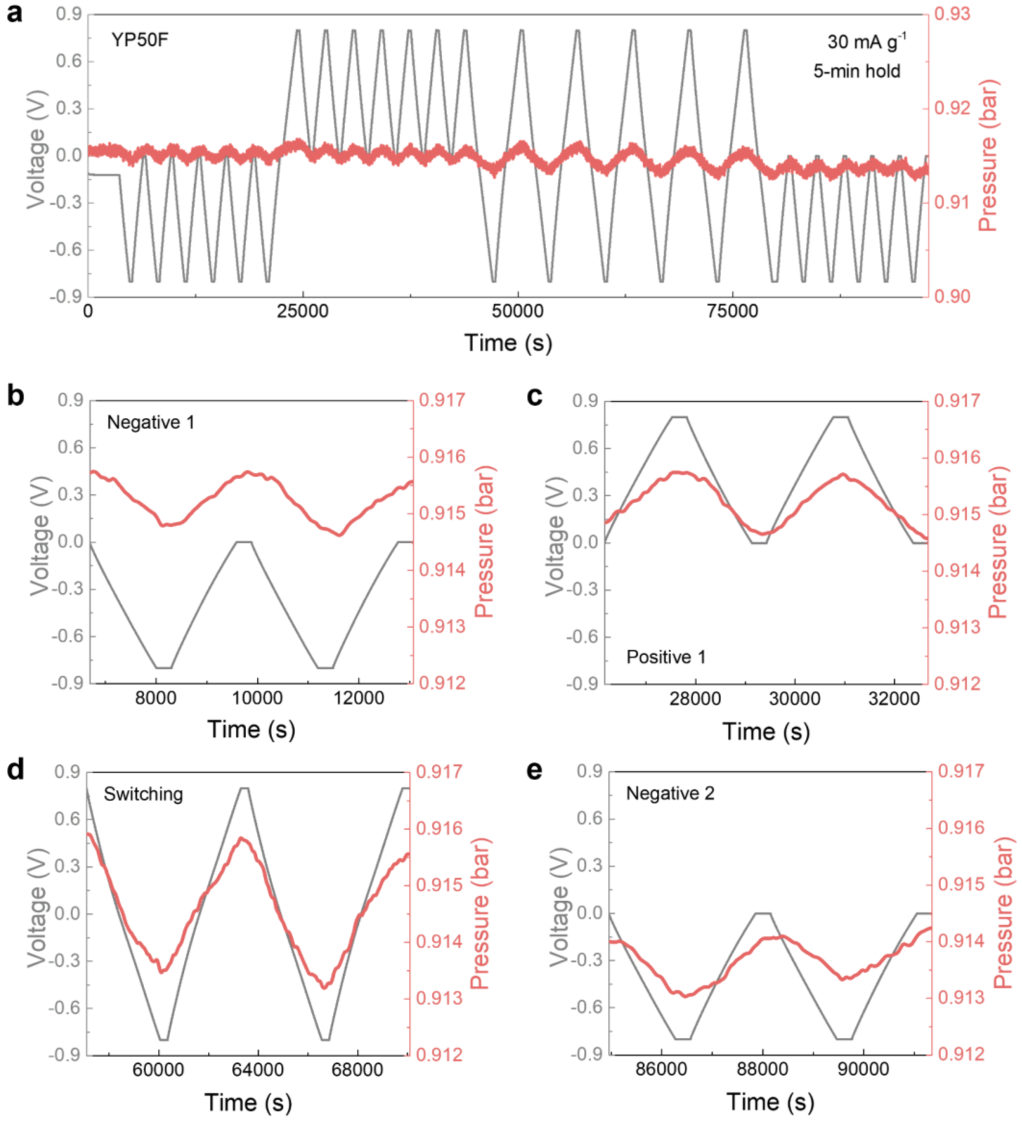

Supplementary Fig. 4. Electrochemical CO_2 capture measurement of YP50F by varying voltage under CO_2. (a) Overall GCD curves (grey) and corresponding pressure curves (red) of the device using YP50F electrodes for CO_2 sorption in negative, positive and switching charging modes (under CO_2, at the current density of 30 mA g^{-1}, with a 5-min voltage hold after the charge or discharge process). **(b-e)** Zoomed GCD curves (grey) and smoothed pressure curves (averaged every 100 sec, red) of the YP50F electrode under CO_2 at the current density of 30 mA g^{-1} in different charging modes, all with 5-min voltage holds. (Negative 1 and 2 represent the first and second negative charging parts, respectively). Notes: In **Supplementary Fig. 4**a, the irreversible pressure decrease during charging and discharging is negligible. Hence, the use of a 5-min voltage hold can reduce the side reactions and maintain the electrochemical CO_2 capture performance.

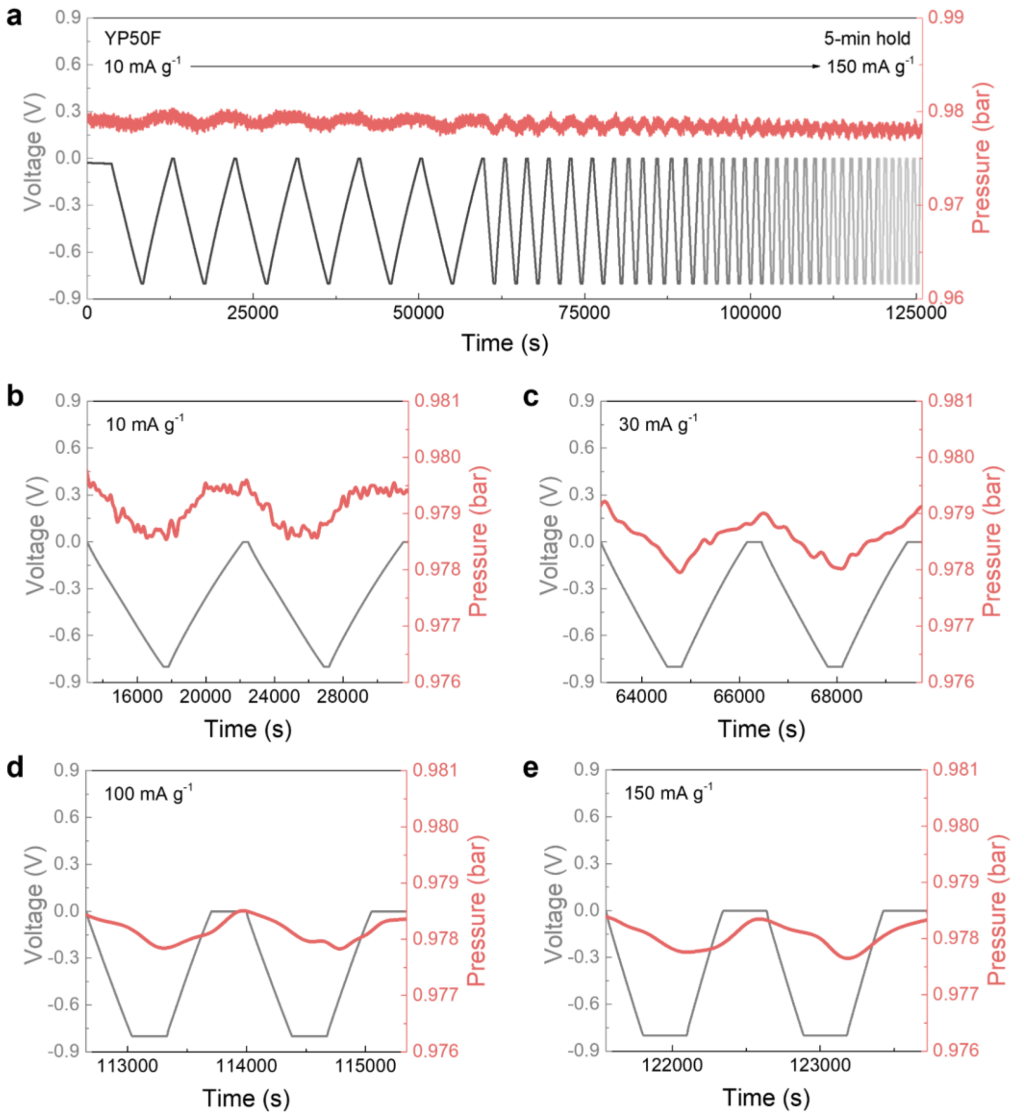

Supplementary Fig. 5. Electrochemical CO_2 capture measurement of YP50F by varying current under CO_2. (a) Overall GCD curves (grey) and corresponding pressure curves (red) of the device using YP50F electrodes for CO_2 sorption in the negative charging mode (under CO_2, at different current densities of 10, 30, 50, 70, 90, 100, 150 mA g^{-1}, with a 5-min voltage hold after the charge or discharge process). **(b-e)** Zoomed GCD curves (grey) and smoothed pressure curves (averaged every 100 sec, red) of the YP50F electrode under CO_2 at different current densities of 10, 30 100 and 150 mA g^{-1} in the negative charging mode, all with 5-min voltage holds.

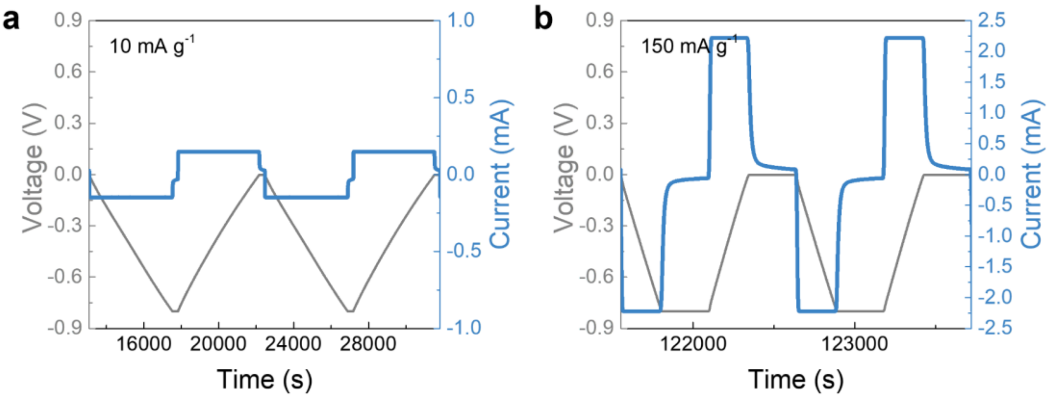

Supplementary Fig. 6. Plots of different currents used for electrochemical CO2 capture measurement of YP50F under CO2. Zoomed GCD curves (grey) and current curves (blue) of the YP50F electrode under CO2 at different current densities of **(a)** 10 and **(b)** 150 mA g–1 in the negative charging mode, with 5-min voltage holds. Notes: The irreversible electrical energy consumption mainly originates from the voltage hold part at ±0.8 V. This is because most energy input during the charging step can be offset by the energy output during the discharging step (**Supplementary Equation (9)**). In addition, the voltage hold at 0 V after the discharging step does not contribute to any irreversible electrical energy recovery (**Supplementary Equation (3)**). Therefore, the main contributor to the irreversible electrical energy consumption is the voltage hold part at 0.8 V after the charging step. When employing a fast charging step with a higher constant current (**Supplementary Fig. 6**b), the following 0.8 V voltage hold leads to higher irreversible electrical energy consumption (**Supplementary Equations (3)** and **(9)**).

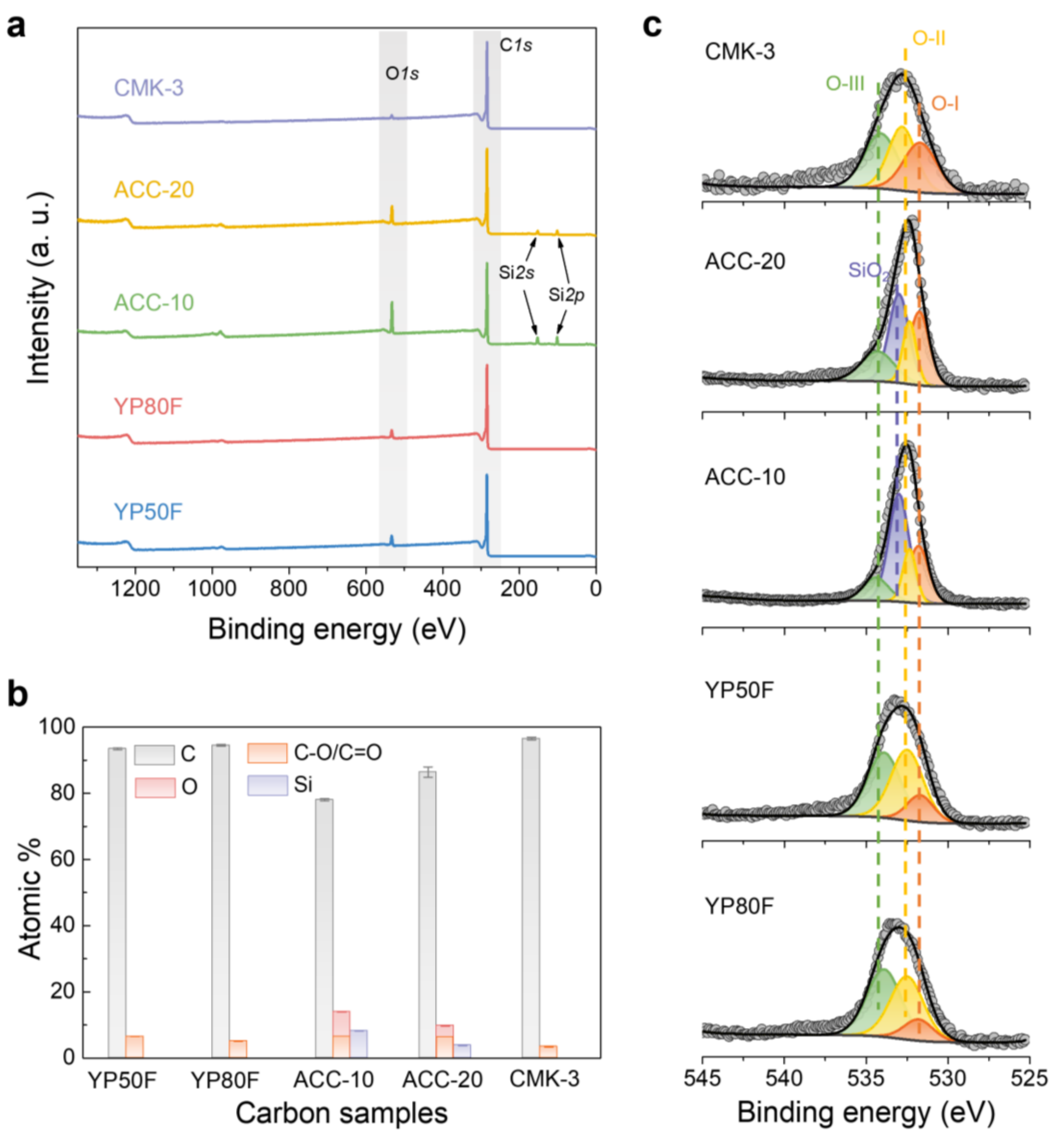

Supplementary Fig. 7. X-ray photoelectron spectroscopy (XPS) measurement of different carbons. (a) XPS survey of YP50F, YP80F, ACC-10, ACC-20 and CMK-3. **(b)** Calculated oxygen atomic amount of YP50F, YP80F, ACC-10, ACC-20 and CMK-3. **(c)** Fitted O_{1s} core-level spectra of YP50F, YP80F, ACC-10, ACC-20 and CMK-3. Notes: O-I represents C=O carbonyl groups, O-II represents C-OH hydroxyl or C-O-C ether groups, and O-III represents O=C-OH carboxyl groups. Error bars represent the standard deviation of atomic percentages of elements at different spots on the same sample.

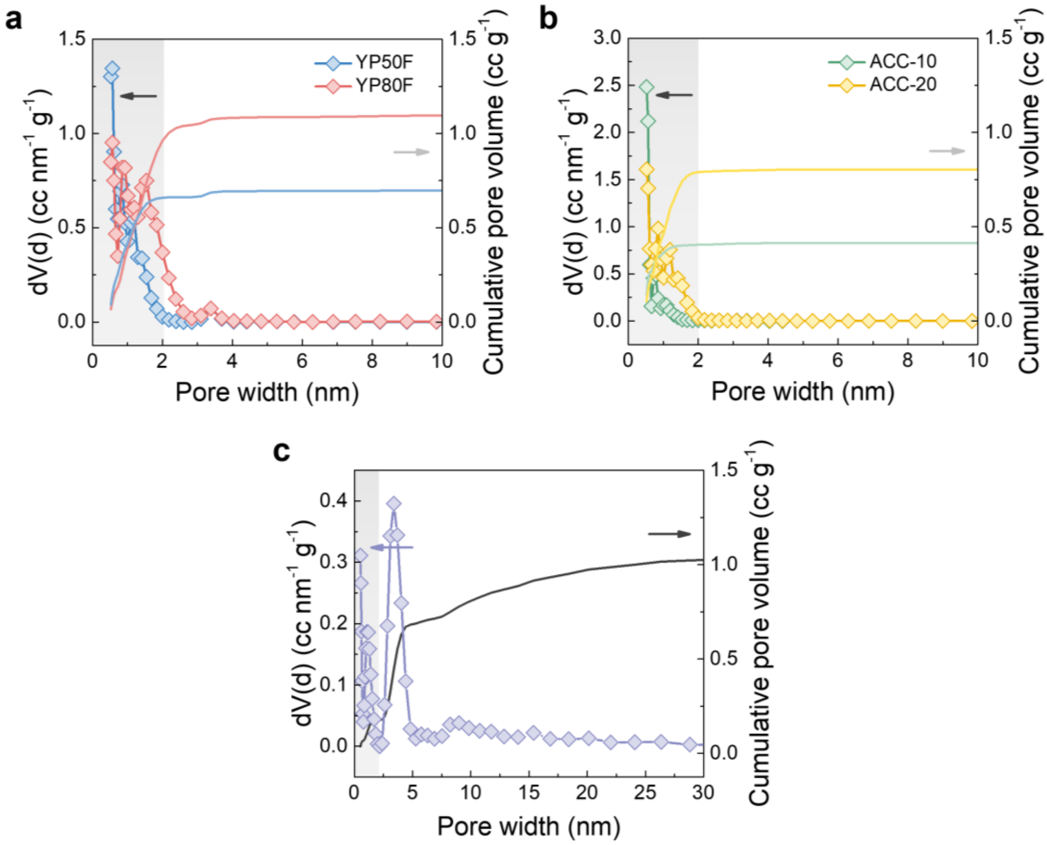

Supplementary Fig. 8. Pore structure analysis of different carbons. Pore size distribution and cumulative pore volume using the quenched solid density functional theory (QSDFT) and slit pore model, of **(a)** YP50F and YP80F, **(b)** ACC-10 and ACC-20, and **(c)** CMK-3.

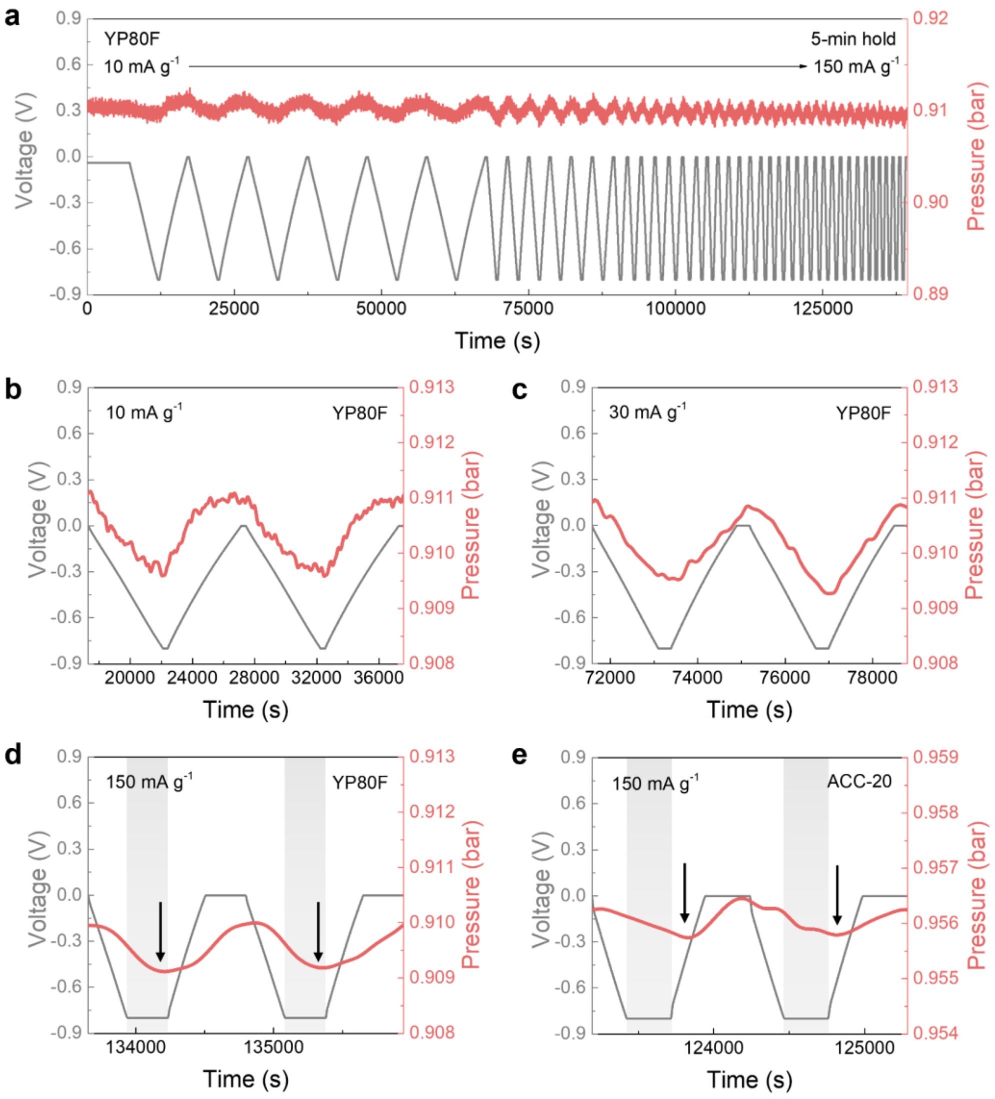

Supplementary Fig. 9. Electrochemical CO_2 capture measurement of YP80F and ACC-20 by varying current under CO_2. (a) Overall GCD curves (grey) and corresponding pressure curves (red) of the device using YP80F electrodes for CO_2 sorption in the negative charging mode (under CO_2, at different current densities of 10, 30, 50, 70, 90, 100, 150 mA g^{-1}, with a 5-min voltage hold after the charge or discharge process). Zoomed GCD curves (grey) and smoothed pressure curves (averaged every 100 sec, red) of the YP80F electrode under CO_2 at the current densities of **(b)** 10 and **(c)** 30 mA g^{-1} in the negative charging mode, with 5-min voltage holds. Zoomed GCD curves (grey) and smoothed pressure curves (averaged every 100 sec, red) of **(d)** YP80F and **(e)** ACC-20 electrodes under CO_2 at the current density 150 mA g^{-1} in the negative charging mode, with 5-min voltage holds (gray regions represent the voltage hold steps, and black arrows represent the maximum and minimum peaks of pressure curves).

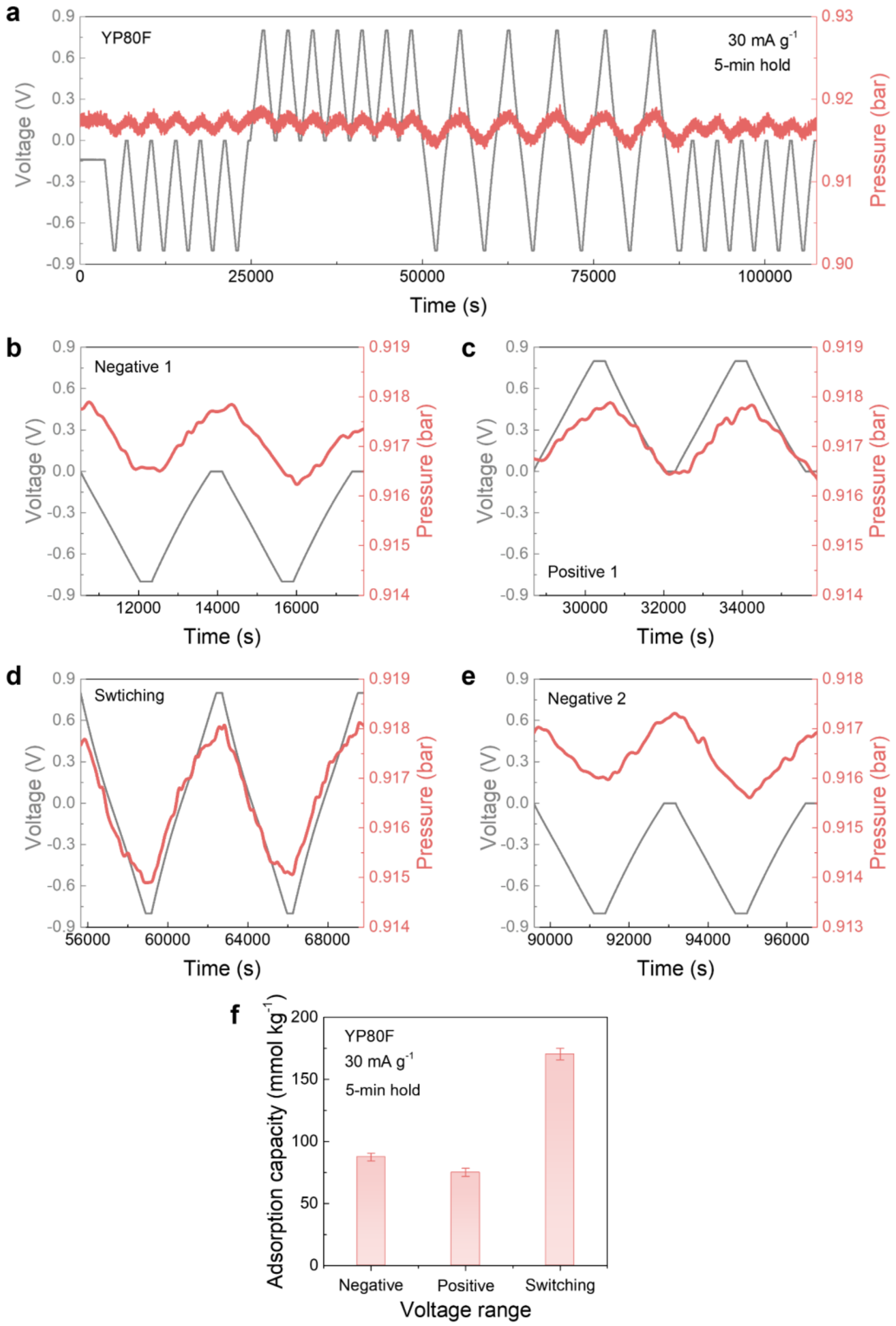

Supplementary Fig. 10. Electrochemical CO_2 capture measurement of YP80F by varying voltage under CO_2. (a) Overall GCD curves (grey) and corresponding pressure curves (red) of the device using YP80F electrodes for CO_2 sorption in negative, positive and switching charging modes (under CO_2, at the current density of 30 mA g^{-1}, with a 5-min voltage hold after the charge or discharge process). **(b-e)** Zoomed GCD curves (grey) and smoothed pressure curves (averaged every 100 sec, red) of the YP80F electrode under CO_2 at the current density

of 30 mA g^{-1} in different charging modes, all with 5-min voltage holds. **(f)** Comparison of the CO_2 adsorption capacity of the YP80F electrode at the current density of 30 mA g^{-1} in the negative, positive and switching charging modes, with 5-min voltage holds. Error bars represent t-test of performance from cycle to cycle at the same charging protocol.

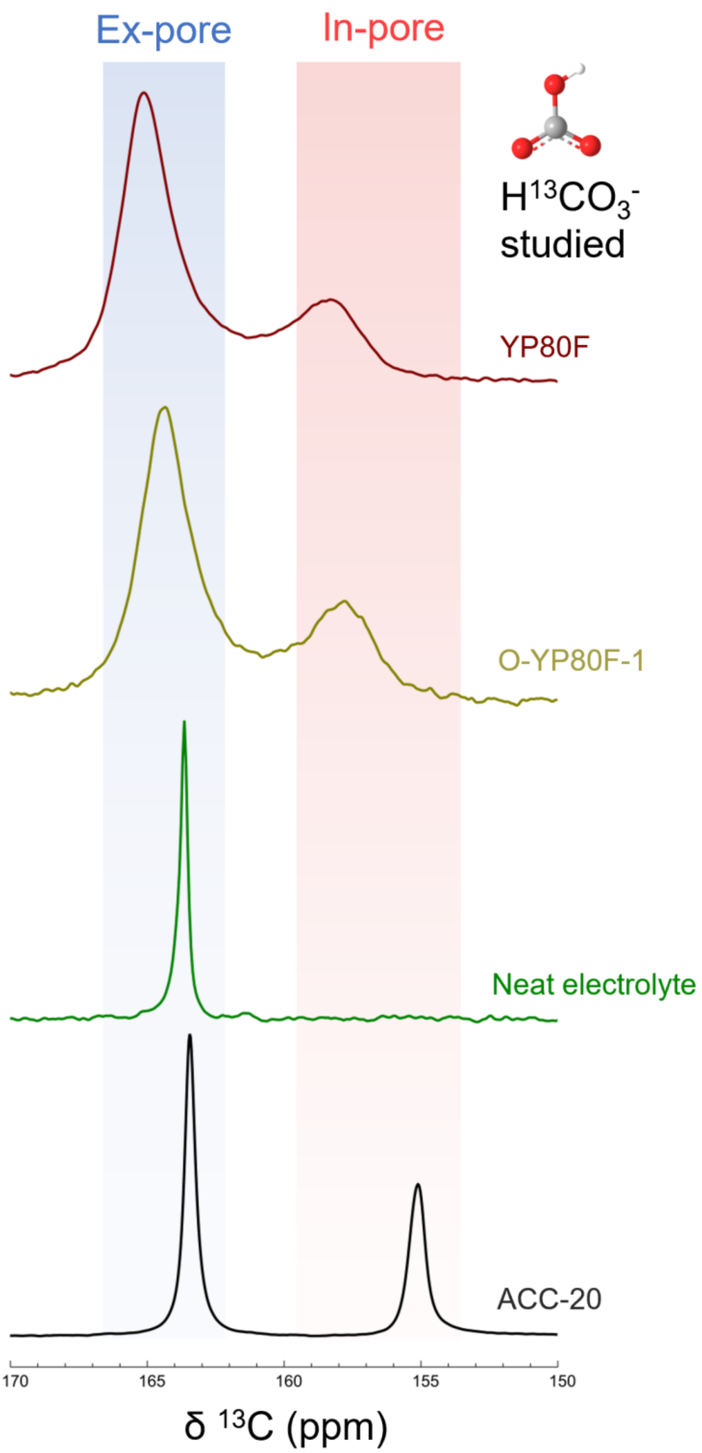

Supplementary Fig. 11. Solid-state nuclear magnetic resonance (NMR) measurement of different carbons. ^{13}C solid-state NMR spectroscopy (9.4 T, 5 kHz MAS) of YP80F, O-YP80F-1 and ACC-20 soaked with 1 M $NaH^{13}CO_3$ (aq) solution as well as neat electrolyte.

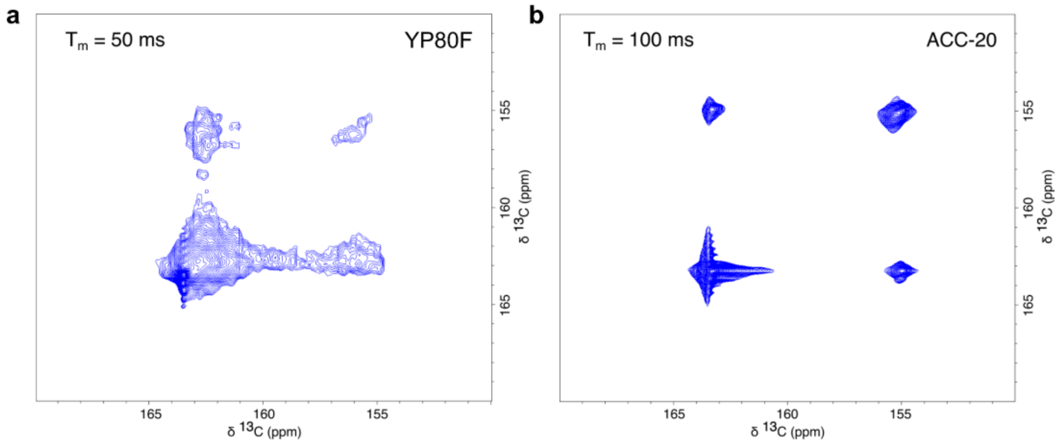

Supplementary Fig. 12. Solid-state nuclear magnetic resonance (NMR) measurement of YP80F and ACC-20. ^{13}C solid-state NMR exchange spectroscopy (EXSY) (9.4 T, 5 kHz MAS) of **(a)** YP80F and **(b)** ACC-20 soaked with 1 M $NaH^{13}CO_3$ (aq) solution. A mixing time of 50 ms was used for YP80F and 100 ms for ACC-20.

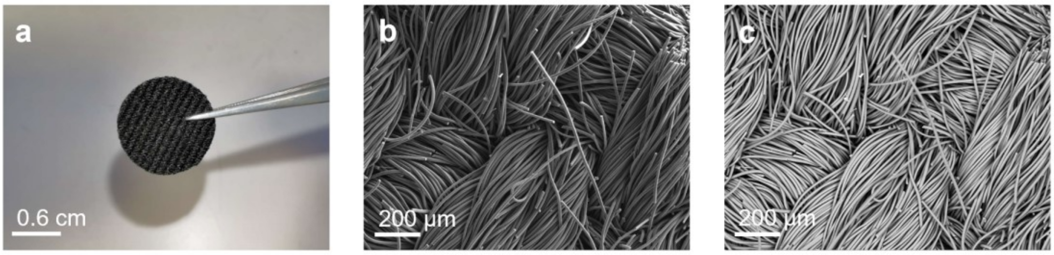

Supplementary Fig. 13. Morphology characterization of ACC-20. **(a)** Photo of an ACC-20 electrode. Scanning electron microscopy (SEM) images of the ACC-20 electrode using **(b)** secondary electrons (SE) and **(c)** backscattered electrons (BSE).

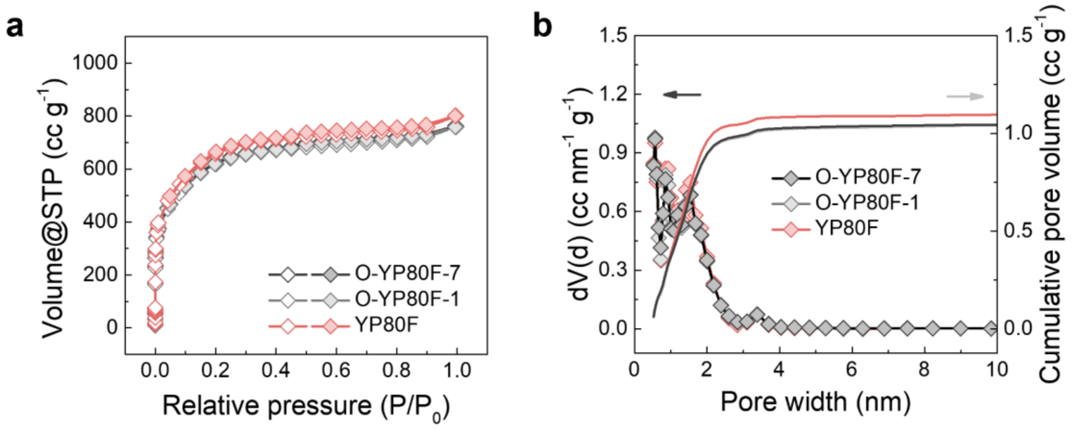

Supplementary Fig. 14. Pore structure analysis of different carbons. (a) N_2 sorption isotherms at 77 K of YP80F, O-YP80F-1, O-YP80-7. **(b)** Pore size distribution and cumulative pore volume using the quenched solid density functional theory (QSDFT) and slit pore model, of YP80F, O-YP80F-1, and O-YP80-7.

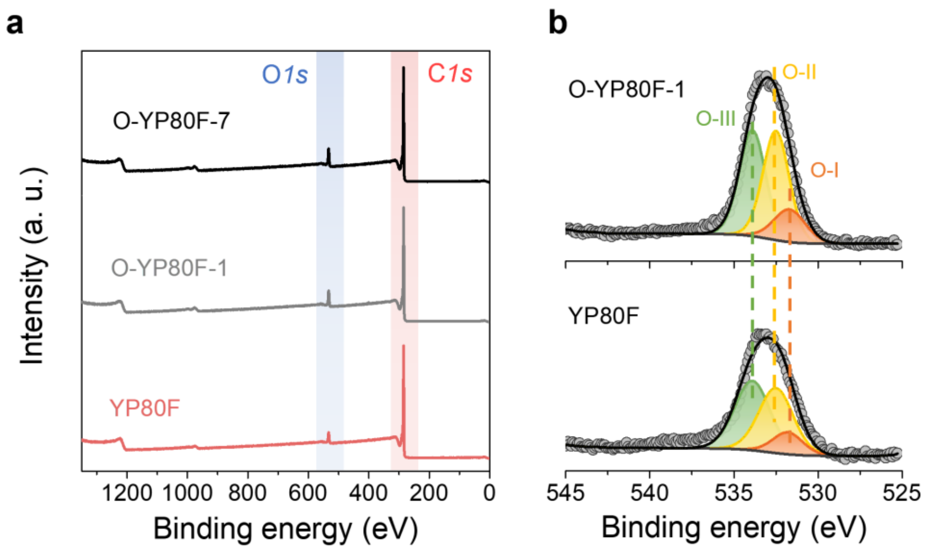

Supplementary Fig. 15. X-ray photoelectron spectroscopy (XPS) measurement of different carbons. (a) XPS survey of YP80F, O-YP80F-1 and O-YP80F-7. **(b)** Fitted O_{1s} core-level spectra of YP80F and O-YP80F-1. Notes: O-I represents C=O carbonyl groups, O-II represents C-OH hydroxyl or C-O-C ether groups, and O-III represents O=C-OH carboxyl groups.

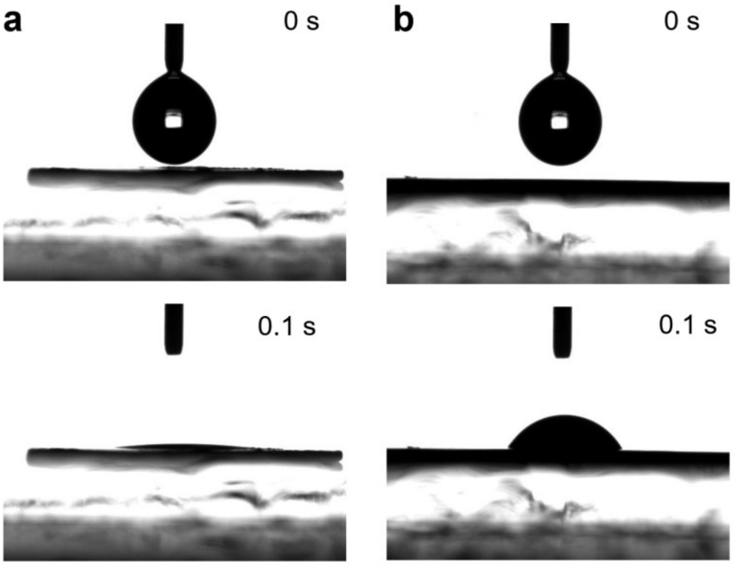

Supplementary Fig. 16. Wettability characterization of different carbons. Contact angle measurement images of **(a)** oxidized YP80F and **(b)** YP80F immediately after triggering using deionized water.

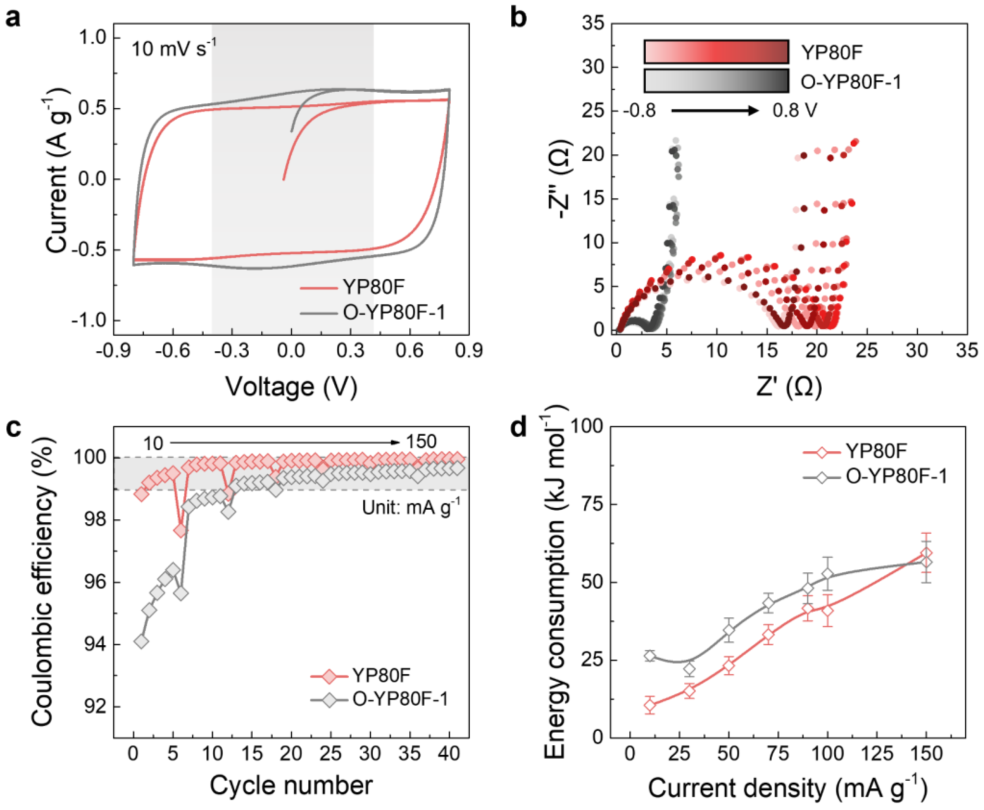

Supplementary Fig. 17. Electrochemical measurement of oxidized YP80F and pristine YP80F under CO_2. (a) Cyclic voltammetry (CV) of oxidized YP80F and YP80F electrodes at the scan rate of 10 mV s^{-1}. **(b)** Electrochemical impedance spectroscopy (EIS) of oxidized YP80F and YP80F electrodes at different charged states using frequencies ranging from 0.01 to 100k Hz. **(c)** Coulombic efficiencies of oxidized YP80F and YP80F electrodes at different current densities. **(d)** Comparison of the electrical energy consumption for CO_2 adsorption of oxidized YP80F and YP80F electrodes at different current densities in the negative charging mode, with 5-min voltage holds. Error bars represent t-test of performance from cycle to cycle at the same charging protocol.

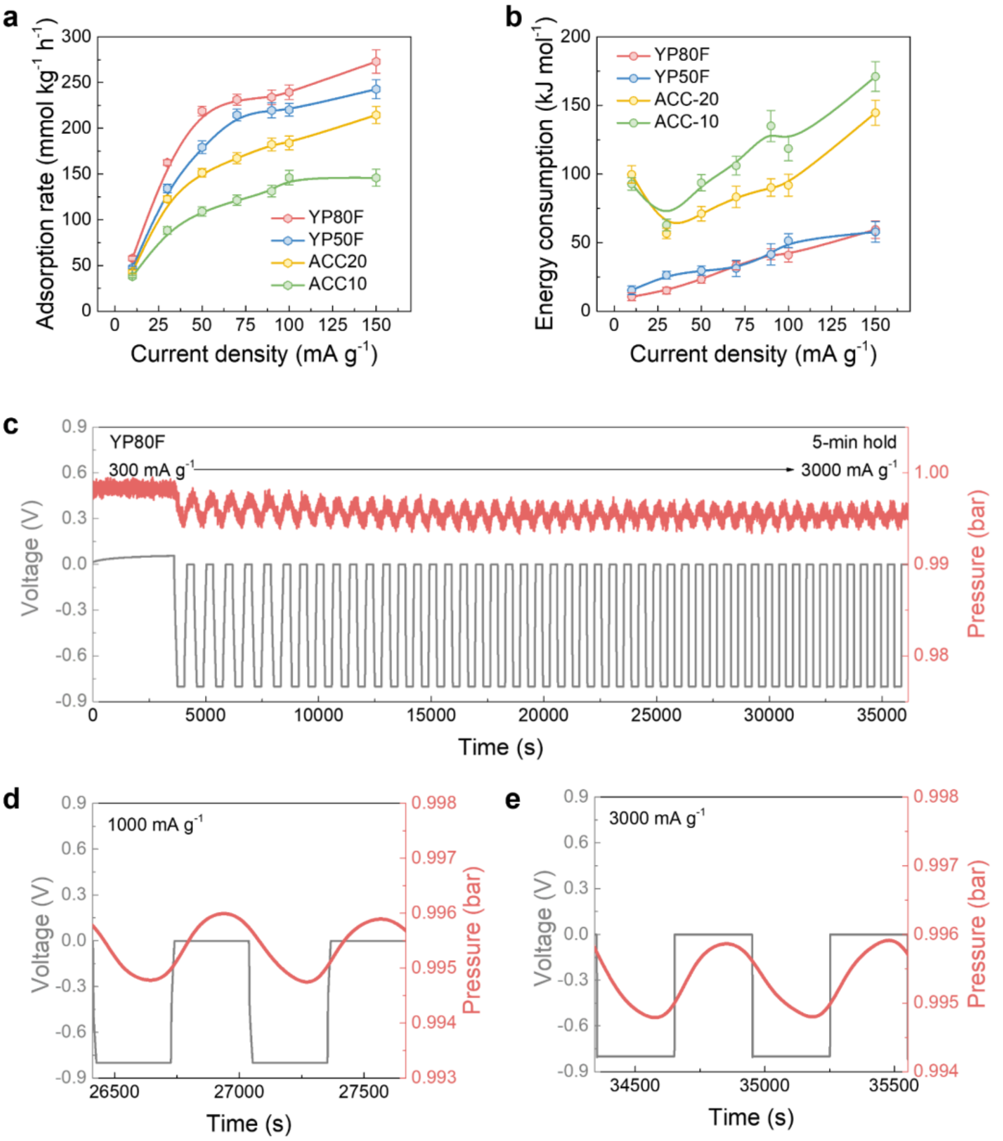

Supplementary Fig. 18. Energy efficiency and capture rate analysis of different carbons, and electrochemical CO_2 capture measurement of YP80F by varying current under CO_2. Comparison of **(a)** the CO_2 adsorption rate (normalized by charging time) and **(b)** electrical energy consumption for CO_2 adsorption of carbon electrodes at different current densities in the negative charging mode, with 5-min voltage holds. **(c)** Overall GCD curves (grey) and corresponding pressure curves (red) of the device using YP80F electrodes for CO_2 sorption in the negative charging mode (under CO_2, at high current densities of 300, 500, 700, 900, 1000, 1500 and 3000 mA g^{-1}, with a 5-min voltage hold after the charge or discharge process). Zoomed GCD curves (grey) and smoothed pressure curves (averaged every 100 sec, red) of the YP80F electrode under CO_2 adsorption at the current densities of **(d)** 1000 and **(e)** 3000 mA g^{-1} in the negative charging mode, with 5-min voltage holds. Error bars represent t-test of performance from cycle to cycle at the same charging protocol. Notes: The observed difference in electrical energy consumption between YP carbons and ACC carbons can be attributed to two factors. Firstly, YP carbons possess a larger CO_2 adsorption capacity compared to ACC carbons. Secondly, more energy is required to overcome the larger polarization of ACC carbons

during the voltage holding time at the charged state because ACC carbons are predominantly microporous and therefore restrict the interfacial contact with electrolytes, in spite of their cloth morphologies with the 3D networks. YP80F exhibits the lowest electrical energy consumption of 10.5 kJ mol_{CO2}^{-1} (10 mA g^{-1}, 0.8 V and 5-min voltage hold). Nevertheless, all carbon electrodes show a similar trend in the CO_2 adsorption rate, with YP80F having the fastest rate of 273 $mmol_{CO2}$ kg^{-1} h^{-1} (150 mA g^{-1}, 0.8 V and 5-min voltage hold). Even though the current density continuously increases, the existence of 5-min voltage holds a dominant role in CO_2 adsorption, thus stabilizing the CO_2 adsorption capacity and rate (**Supplementary Fig. 18**c-e).

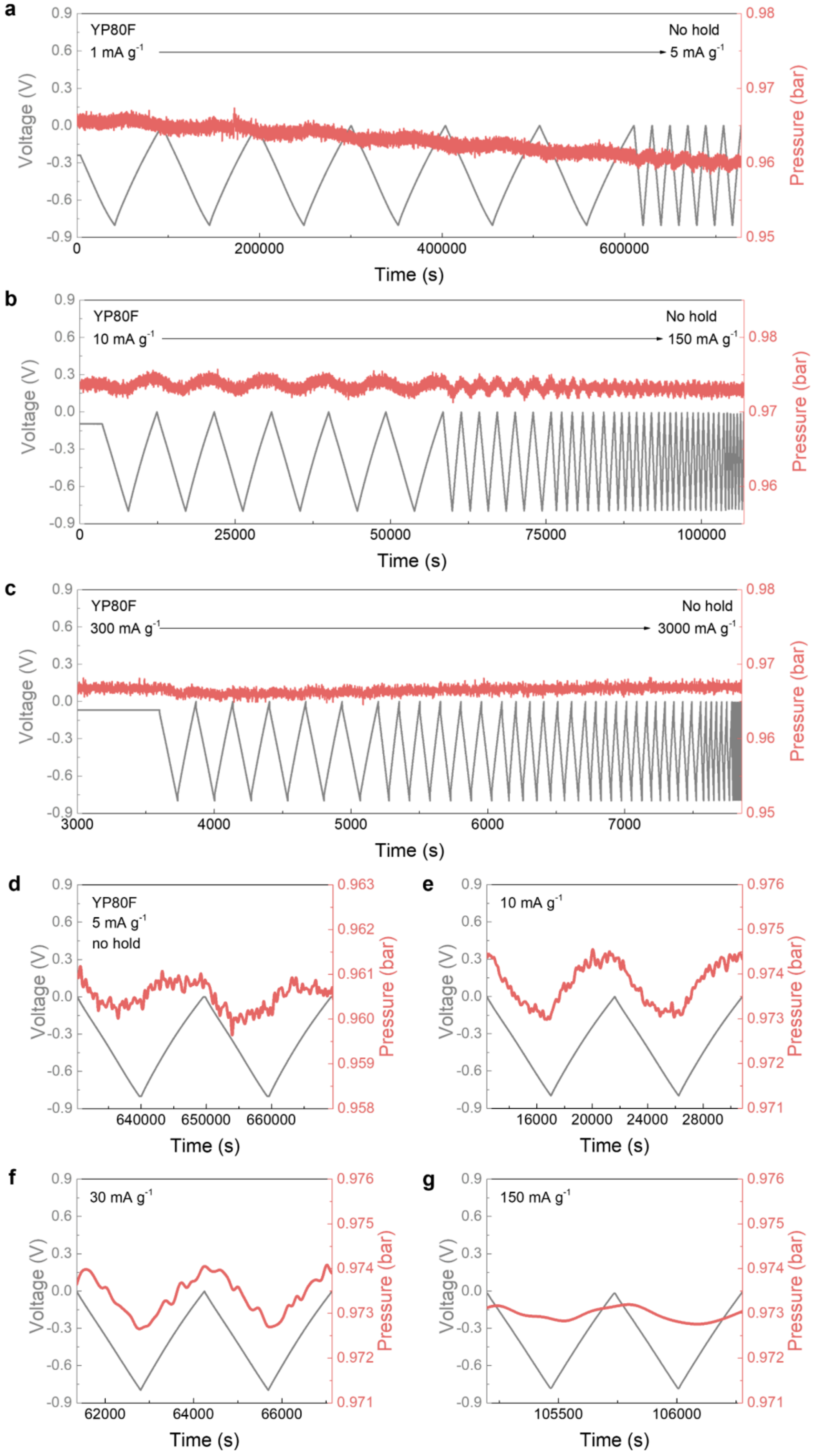

Supplementary Fig. 19. Electrochemical CO_2 capture measurement of YP80F by varying

current under CO_2. Overall GCD curves (grey) and corresponding pressure curves (red) of the device using YP80F electrodes for CO_2 sorption in the negative charging mode **(a)** at low current densities of 1 and 5 mA g^{-1}, **(b)** at middle current densities of 10, 30, 50, 70, 90, 100 and 150 mA g^{-1}, and **(c)** at high current densities of 300, 500, 700, 900, 1000, 1500 and 3000 mA g^{-1}, all under CO_2 and without voltage hold. **(d-g)** Zoomed GCD curves (grey) and smoothed pressure curves (averaged every 100 sec, red) of the YP80F electrode under CO_2 at the current densities of 5, 10, 30 and 150 mA g^{-1} in the negative charging mode, all without voltage hold.

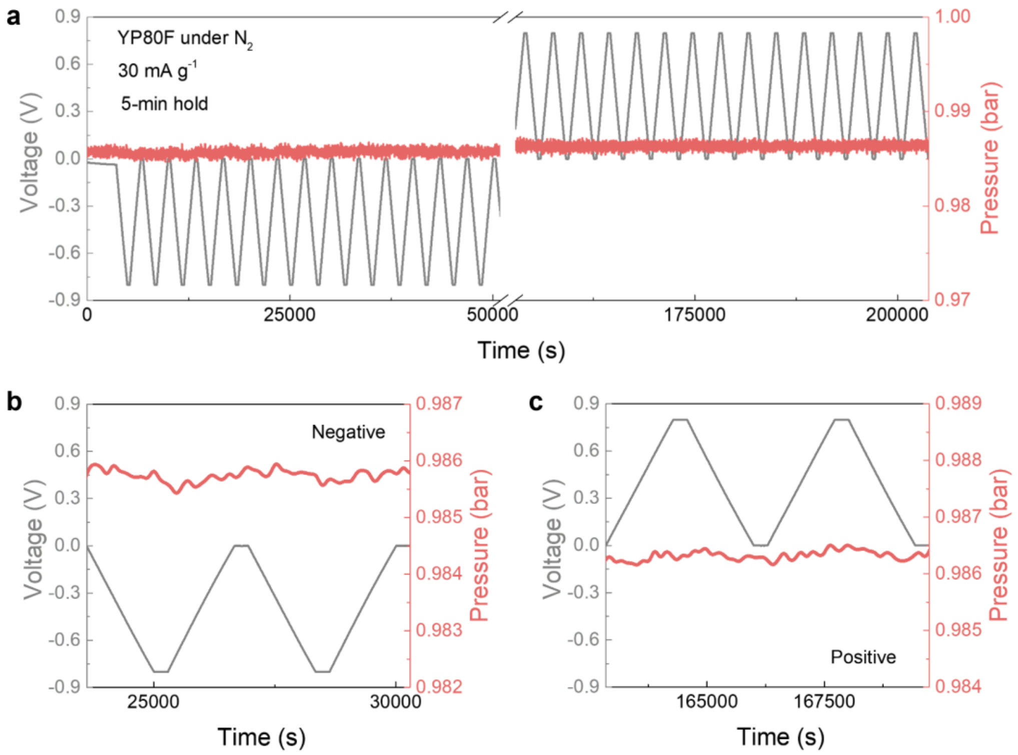

Supplementary Fig. 20. Electrochemical measurement of YP80F under N_2. (a) Overall GCD curves (grey) and corresponding pressure curves (red) of the device using YP80F electrodes under N_2 at the current densities of 30 mA g^{-1} in the negative and positive charging modes, with 5-min voltage holds. Zoomed GCD curves (grey) and smoothed pressure curves (averaged every 100 sec, red) of the YP80F electrode under N_2 at the current densities of 30 mA g^{-1} in **(b)** the negative charging mode and **(c)** the positive charging mode, with 5-min voltage holds. Notes: In **Supplementary Fig. 20**a, the averaged Coulombic efficiencies in the positive and negative charging modes are both above 99.5%, which indicates the high reversibility of the electrochemical processes under N_2.

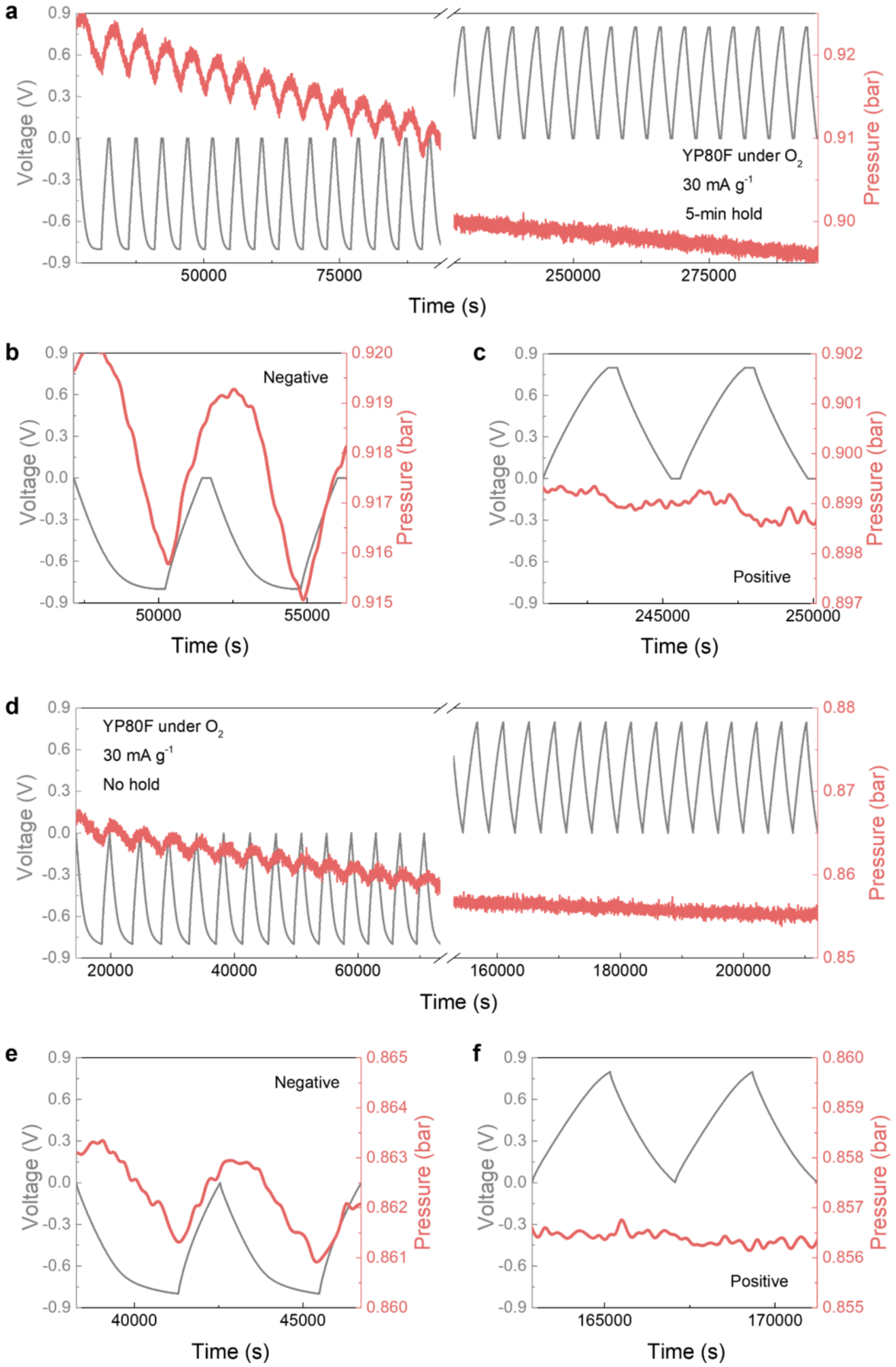

Supplementary Fig. 21. Electrochemical measurement of YP80F under O_2. (a) Overall GCD curves (grey) and corresponding pressure curves (red) of the device using YP80F electrodes under O_2 at the current densities of 30 mA g^{-1} in the negative and positive charging modes, with 5-min voltage holds. Zoomed GCD curves (grey) and smoothed pressure curves (averaged every 100 sec, red) of the YP80F electrode under O_2 at the current densities of 30

mA g^{-1} in **(b)** the negative charging mode and **(c)** the positive charging mode, with 5-min voltage holds. **(d)** Overall GCD curves (grey) and corresponding pressure curves (red) of the device using YP80F electrodes under O_2 at the current densities of 30 mA g^{-1} in the negative and positive charging modes, without voltage hold. Zoomed GCD curves (grey) and smoothed pressure curves (averaged every 100 sec, red) of the YP80F electrode under O_2 at the current densities of 30 mA g^{-1} in **(e)** the negative charging mode and **(f)** the positive charging mode, without voltage hold. Notes: In **Supplementary Fig. 21**a, the average Coulombic efficiency in the positive charging mode is 85%, while in **Supplementary Fig. 21**d, the average Coulombic efficiency in the positive charging mode is 87%, both indicating the existence of side reactions between O_2 and the working electrode. However, compared with the negative charging mode with an average Coulombic efficiency below 85%, the positive charging mode is promising to realize the selectivity over N_2 and O_2. In practical applications, the O_2 volume percentage is much lower than 100%. The removal of the voltage hold further reduces the level of side reactions in the positive charging mode. The difference in the Coulombic efficiencies of the negative charging mode and the positive charging mode provides evidence that oxygen reduction is diffusion-limited in the positive charging mode. This occurs because in the negative charging mode, the working electrode directly carries electrons and allows the oxygen reduction to happen, while in the positive charging mode, the counter electrode carries electrons and is covered by the separator, electrolyte, and the working electrode, which provide diffusive barriers to O_2 transport.

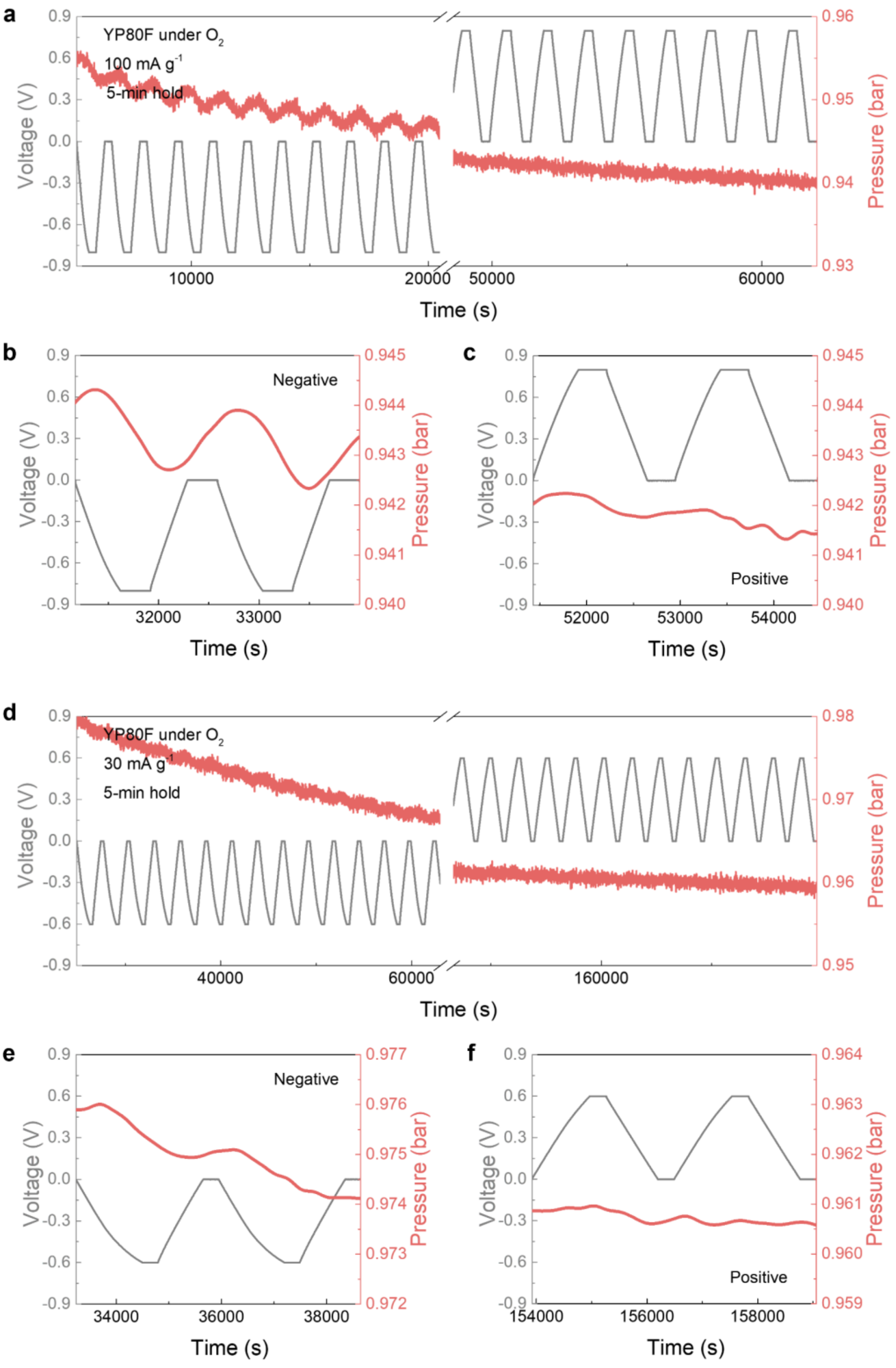

Supplementary Fig. 22. Electrochemical measurement of YP80F under O_2. (a) Overall GCD curves (grey) and corresponding pressure curves (red) of the device using YP80F electrodes under O_2 at the current density of 100 mA g^{-1} in negative and positive charging modes (±0.8 V), with 5-min voltage holds. Zoomed GCD curves (grey) and smoothed pressure curves (averaged every 100 sec, red) of the YP80F electrode under O_2 at the current densities

of 100 mA g^{-1} in **(b)** the negative charging mode and **(c)** the positive charging mode, with 5-min voltage holds. **(d)** Overall GCD curves (grey) and corresponding pressure curves (red) of the device using YP80F electrodes under O_2 at the current density of 30 mA g^{-1} in negative and positive charging modes (± 0.6 V), with 5-min voltage holds. Zoomed GCD curves (grey) and smoothed pressure curves (averaged every 100 sec, red) of the YP80F electrode under O_2 at the current densities of 30 mA g^{-1} in **(e)** the negative charging mode and **(f)** the positive charging mode, with 5-min voltage holds. Notes: In **Supplementary Fig. 22**a, the average Coulombic efficiency in the positive charging mode is 94%, while in **Supplementary Fig. 22**d, the average Coulombic efficiency in the positive charging mode is 92%, which both exhibit the improved reversibility of the electrochemical processes. Therefore, the increase in the current density and the decrease in the voltage range further reduce the level of side reactions in the positive charging mode.

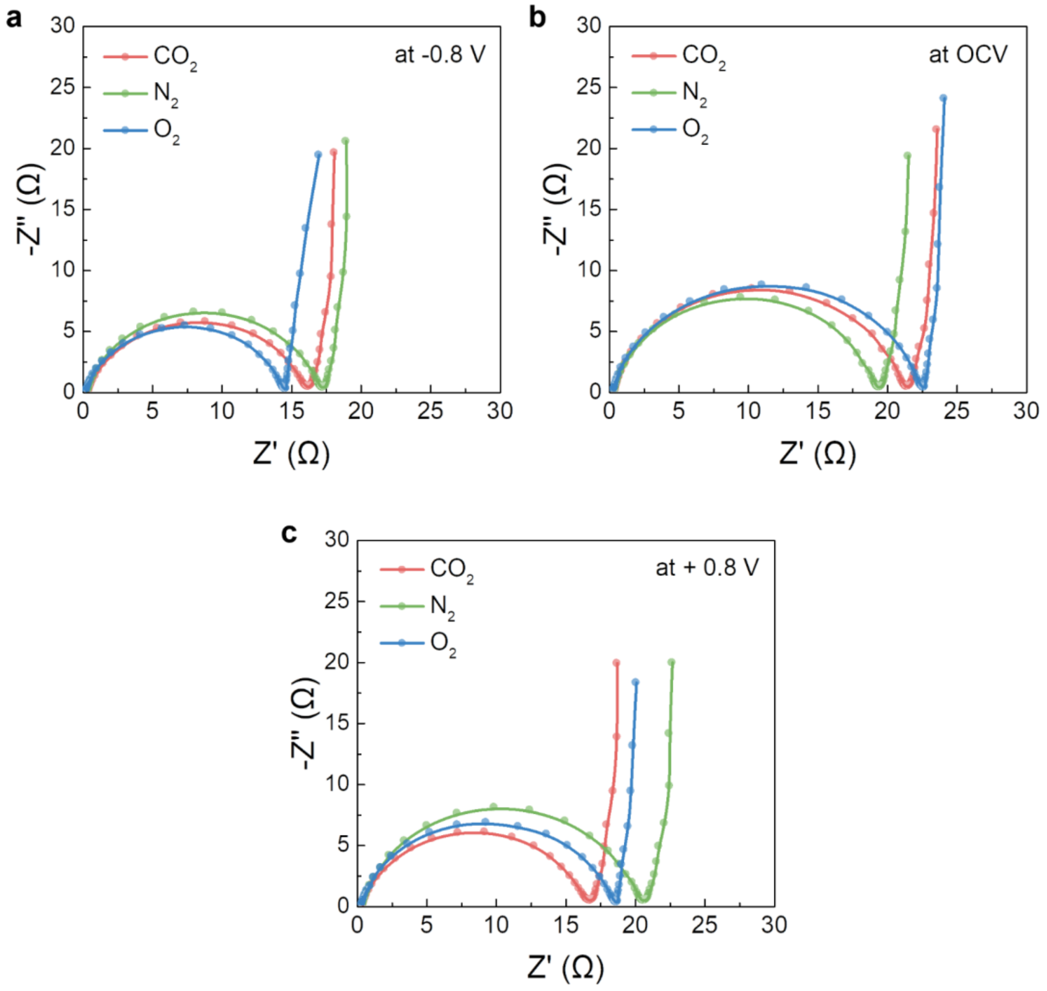

Supplementary Fig. 23. Comparison of electrochemical behaviors of YP80F under different gases. EIS plots of YP80F and YP80F under CO_2, N_2 and O_2 at **(a)** –0.8 V, **(b)** open-circuit voltage (OCV, *i.e.,* 0 V), and **(c)** +0.8 V, using frequencies ranging from 0.01 to 100k Hz. Notes: All these measurements were conducted in gas cells. Except for the EIS at –0.8 V under O_2 (**Supplementary Fig. 23**a), all other EIS exhibits a traditional capacitor-type Nyquist plot,[6,7] which is correlated with the obvious faradic current at –0.8 V under O_2 in **Fig. 4**c and the non-linear charging curves in **Supplementary Fig. 21** in the negative charging mode. Therefore, the positive charging mode was selected to realize the selective separation of CO_2 from mixed gases.

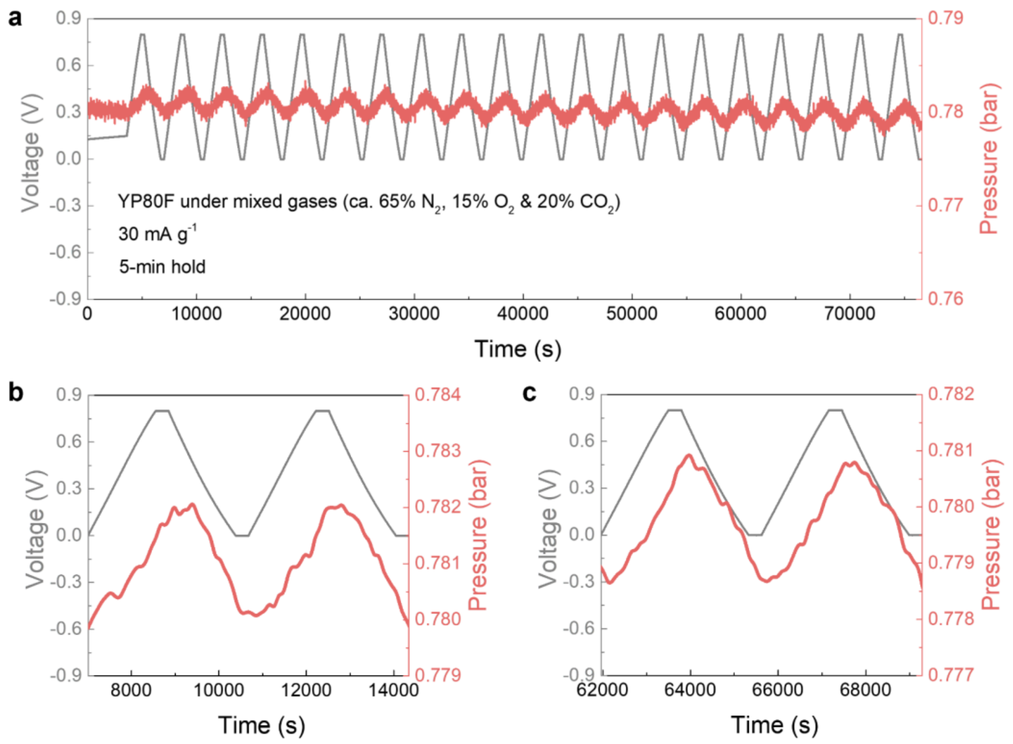

Supplementary Fig. 24. Electrochemical CO_2 capture measurement of YP80F under a gas mixture (approximately 20% CO_2, 15% O_2 and 65% N_2 at around 0.8 bar total pressure). **(a)** Overall GCD curves (grey) and corresponding pressure curves (red) of the device using YP80F electrodes for CO_2 sorption in the positive charging mode (under mixed gases, at the current density of 30 mA g^{-1}, with a 5-min voltage hold after the charge or discharge process). **(b)** and **(c)** Zoomed GCD curves (grey) and smoothed pressure curves (averaged every 100 sec, red) of the YP80F electrode under mixed gases at the current densities of 30 mA g^{-1} in the positive charging mode, with 5-min voltage holds. Notes: Based on the excellent selectivity (**Fig. 4**d and e), the reversible pressure curve change under mixed gases in **Supplementary Fig. 24** is assumed to correspond to the CO_2 adsorption and desorption processes. Furthermore, the experiment employed the 5-min voltage hold for each cycle, and after 20 cycles at 30 mA g^{-1}, the irreversible pressure decrease is negligible. Together with the high Coulombic efficiency (over 99.8%) under this gas mixture, this result further indicates the excellent selectivity and CO_2 capture reversibility of the device with the presence of O_2 and N_2. Based on our measurements using a gas mixture, we observe that the absolute CO_2 amount in the gas chamber is approximately 0.1 mmol, with a composition of around 20% CO_2, 15% O_2, and 65% N_2. During each cycle of our electrochemical CO_2 capture process, it captures approximately 0.002 mmol of CO_2. This corresponds to a reduction of about 2% of the CO_2 concentration per cycle. Larger reductions would be expected for cells with larger electrodes (while keeping the gas volume constant).

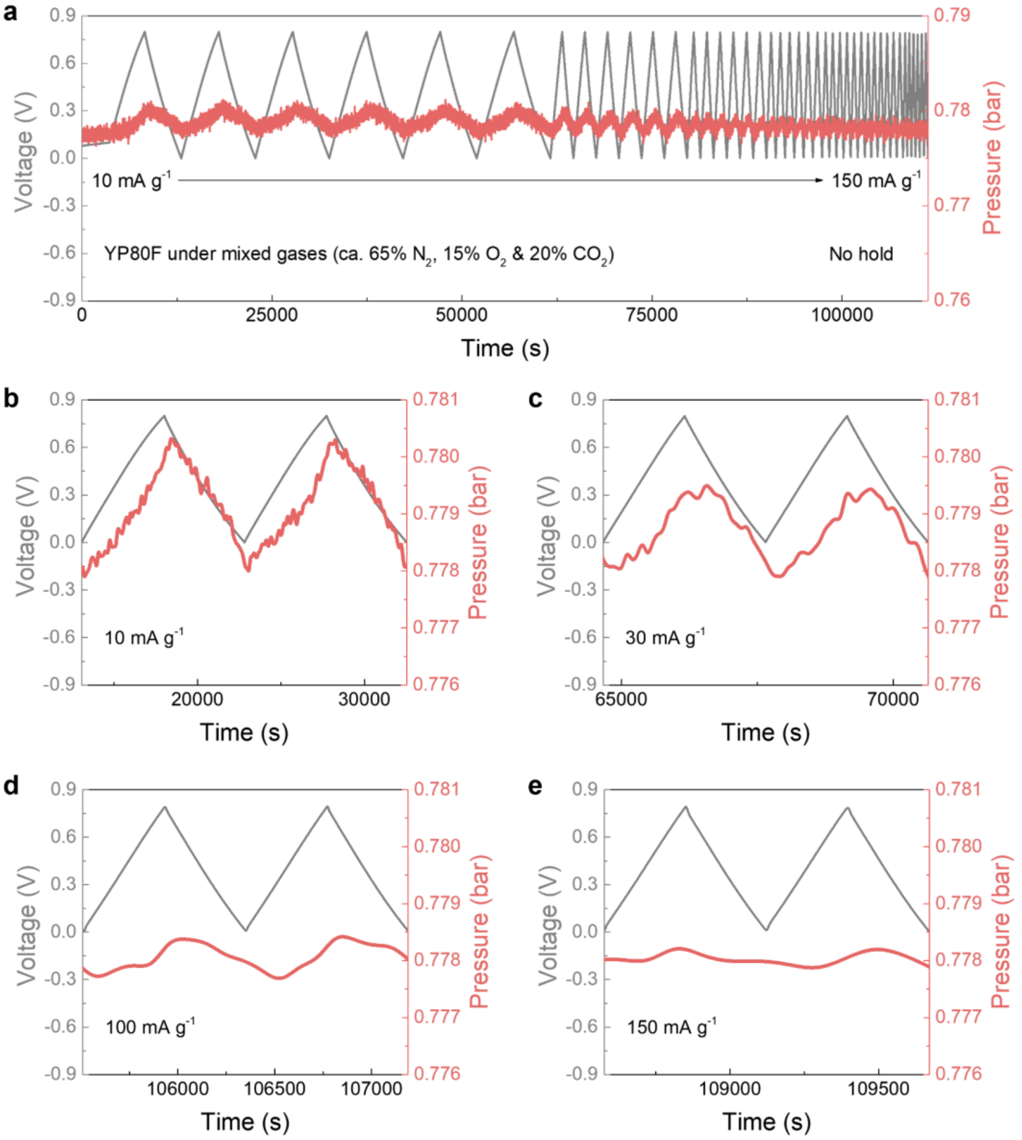

Supplementary Fig. 25. Electrochemical CO_2 capture measurement of YP80F by varying current under a gas mixture (approximately 20% CO_2, 15% O_2 and 65% N_2 at around 0.8 bar total pressure). (a) Overall GCD curves (grey) and corresponding pressure curves (red) of the device using YP80F electrodes for CO_2 sorption in the positive charging mode (under mixed gases, at the current densities of 10, 30, 50, 70, 90, 100 and 150 mA g^{-1}, without voltage hold after the charge or discharge process). **(b-e)** Zoomed GCD curves (grey) and smoothed pressure curves (averaged every 100 sec, red) of the YP80F electrode under mixed gases at the current densities of 10, 30, 100 and 150 mA g^{-1} in the positive charging mode, all without voltage hold.

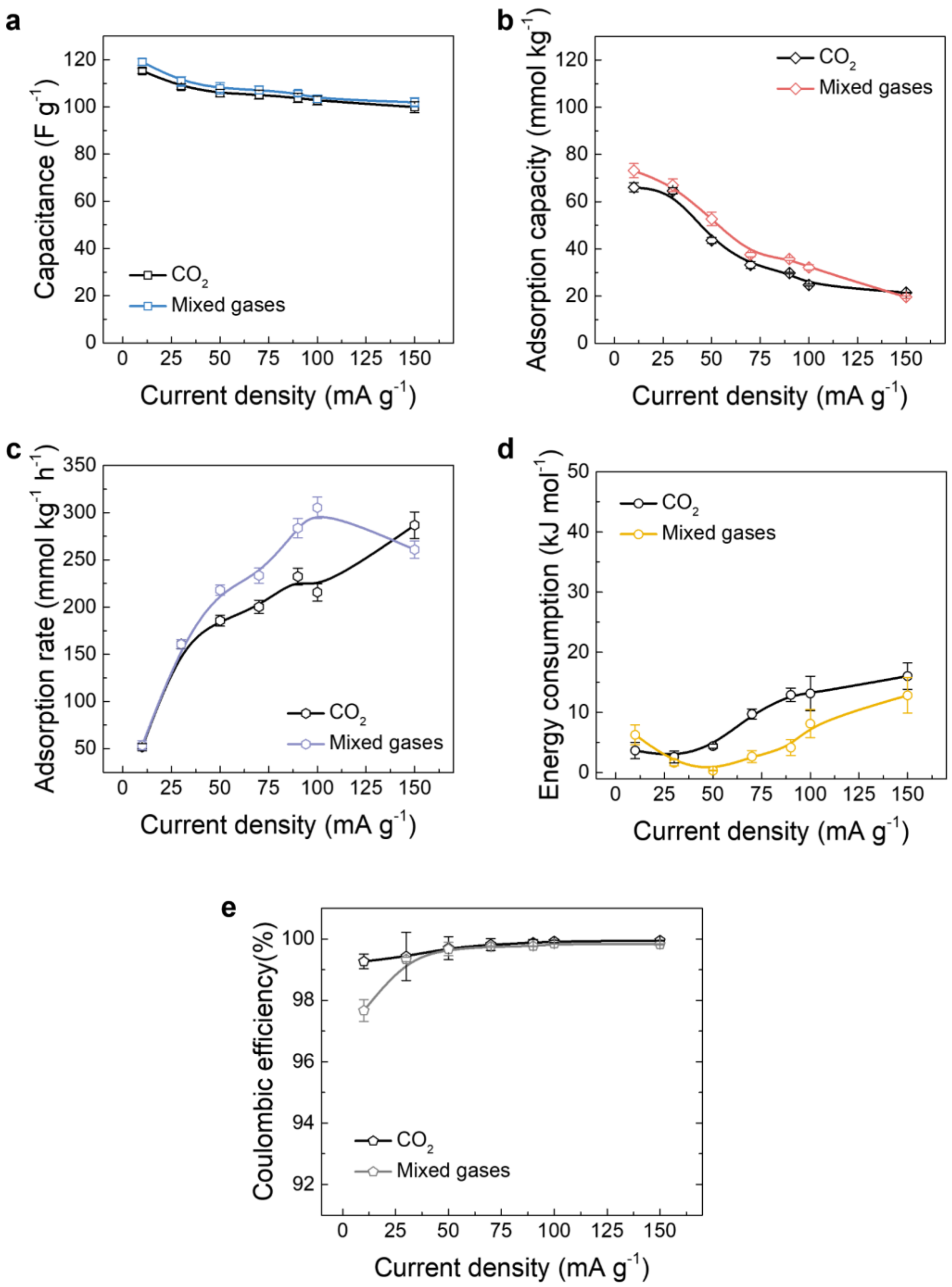

Supplementary Fig. 26. Comparison of electrochemical CO_2 capture performance of YP80F under CO_2 and the gas mixture (approximately 20% CO_2, 15% O_2 and 65 %N_2 at around 0.8 bar total pressure). Comparison of **(a)** the discharge capacitance, **(b)** CO_2 adsorption capacity, **(c)** adsorption rate (normalized by charging time), **(d)** electrical energy consumption and **(e)** Coulombic efficiency of the YP80F electrode under CO_2 in the negative charging mode and under the mixed gas condition in the positive charging mode at different current densities of 10, 30, 50, 70, 90, 100 and 150 mA g^{-1}, all without voltage hold. Error bars represent t-test of performance from cycle to cycle at the same charging protocol.

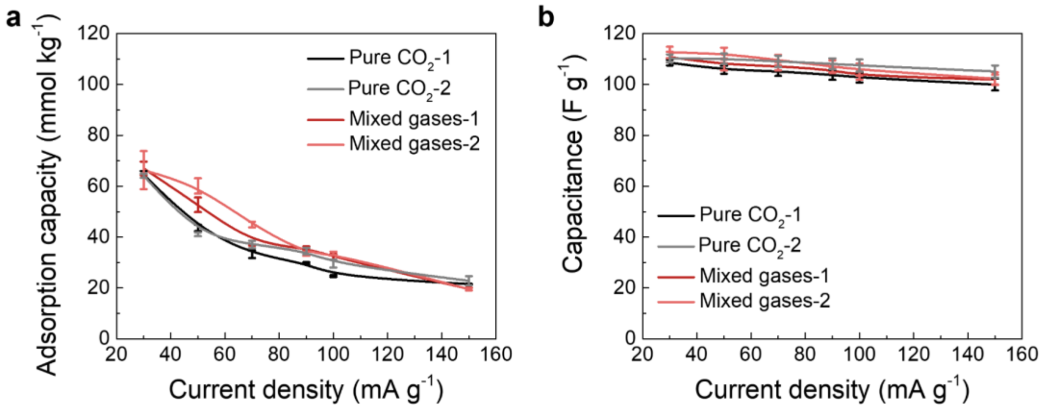

Supplementary Fig. 27. Tests of the reproducibility of electrochemical CO_2 measurements under pure CO_2 and mixed gas conditions (approximately 20% CO_2, 15% O_2 and 65% N_2 at around 0.8 bar total pressure). Comparison of **(a)** CO_2 adsorption capacity and **(b)** the discharge capacitance of the YP80F electrode under CO_2 in the negative charging mode and mixed gas conditions in the positive charging mode at different current densities of 30, 50, 70, 90, 100 and 150 mA g^{-1}, all without voltage hold. Error bars represent t-test of performance from cycle to cycle at the same charging protocol. Note: Cell 1 under the mixed gas condition (mixed gases-1) was dosed under 0.5 bar CO_2 for 5 times before the dosing of 1 bar air, while Cell 2 under the mixed gas condition (mixed gases-2) was dosed under 0.2 bar for 4 times and then under 0.5 bar CO_2 for 1 time. The different gas dosing protocols do not have a significant effect on the electrochemical CO_2 capture performance. Pure CO_2-1 and pure CO_2-2 represent two repeat experiments under pure CO_2. In **Supplementary Fig. 26**, we respectively selected pure CO_2-1 and mixed gases-1 as the representative cells under pure CO_2 and mixed gas conditions for comparison.

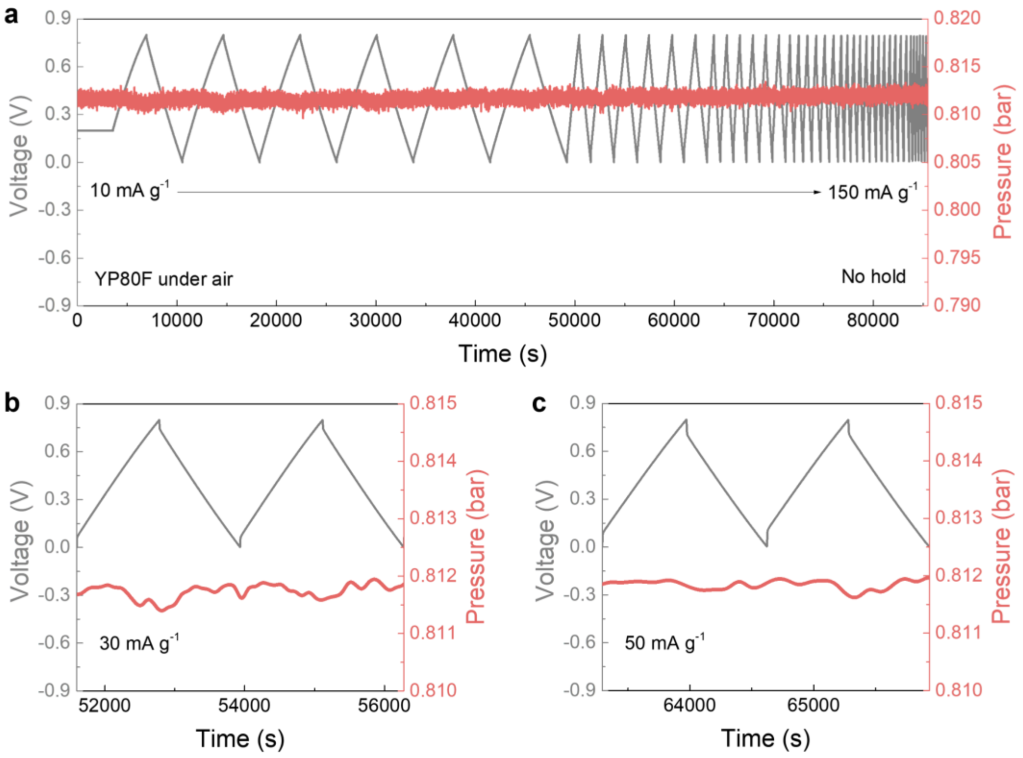

Supplementary Fig. 28. Electrochemical CO_2 capture measurement of YP80F by varying current under ambient air conditions at 0.8 bar. (a) Overall GCD curves (grey) and corresponding pressure curves (red) of the device using YP80F electrodes for CO_2 sorption in the positive charging mode (under air, at the current densities of 10, 30, 50, 70, 90, 100 and 150 mA g^{-1}, without voltage hold after the charge or discharge process). **(b-c)** Zoomed GCD curves (grey) and smoothed pressure curves (averaged every 100 sec, red) of the YP80F electrode under mixed gases at the current densities of 30 and 50 mA g^{-1} in the positive charging mode, all without voltage hold.

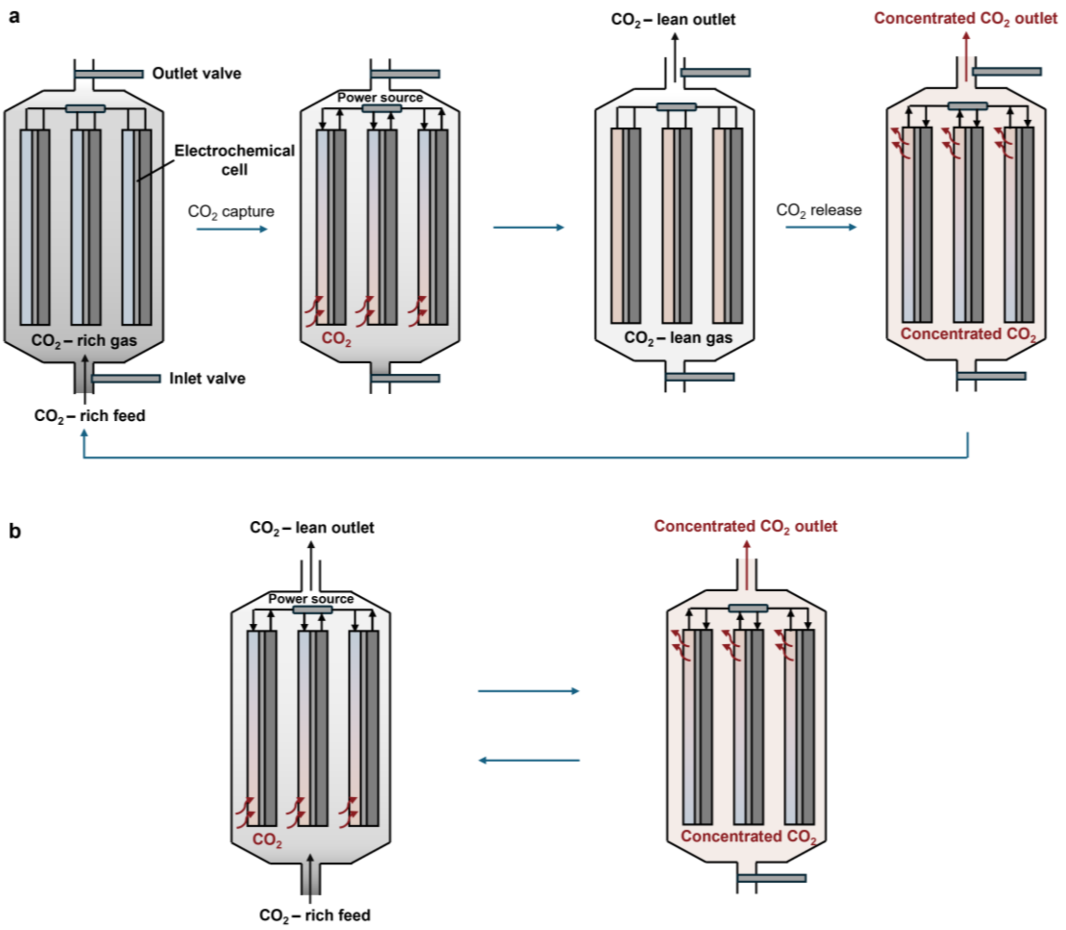

Supplementary Fig. 29. **Possible engineering design of batch-mode electrochemical CO_2 capture with supercapacitors for practical applications.**[8] Schematic illustration of the loop of batch-mode electrochemical CO_2 capture with supercapacitors under **(a)** static gas atmosphere and **(b)** flow gas condition. Notes: The engineering design as shown in **Supplementary Fig. 29**a features two valves controlling the gas inlet and outlet, enabling batch-mode electrochemical CO_2 capture in a static gas environment.

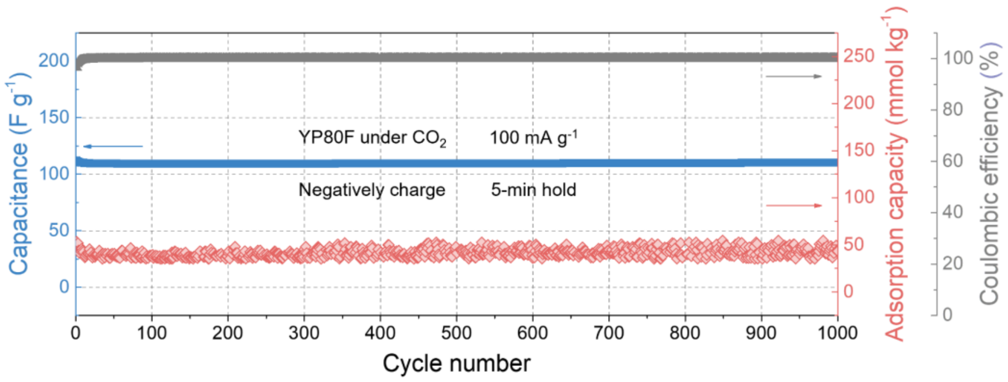

Supplementary Fig. 30. Prolonged cycling measurement of YP80F under pure CO_2. Long cycling performance under CO_2 including the discharge capacitance, CO_2 adsorption capacity and Coulombic efficiency of the YP80F electrode at the current density of 100 mA g^{-1} in the negative charging mode, with 5-min voltage holds.

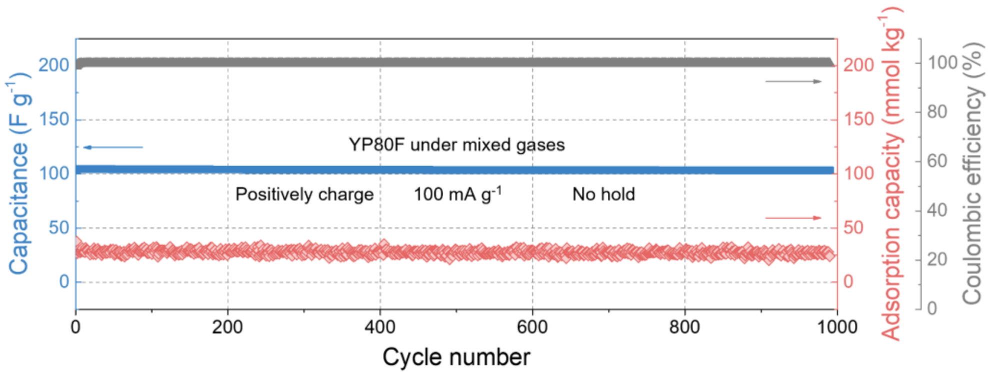

Supplementary Fig. 31. Prolonged cycling measurement of YP80F under a gas mixture (approximately 20% CO_2, 15% O_2 and 65% N_2 at around 0.8 bar total pressure). Long cycling performance under mixed gases including the discharge capacitance, CO_2 adsorption capacity and Coulombic efficiency of the YP80F electrode at the current density of 100 mA g^{-1} in the positive charging mode, without voltage hold.

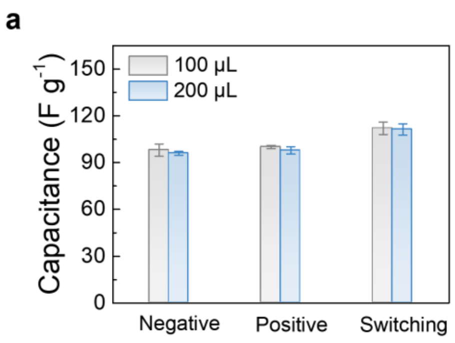

Supplementary Fig. 32. Effect of electrolyte amount on electrochemical CO_2 capture. Comparison of **(a)** the discharge capacitance and **(b)** CO_2 adsorption capacity of the YP50F electrode under CO_2 at the current density of 30 mA g^{-1} in different charging modes (*i.e.,* negative, positive and switching charging modes), with a voltage hold time of 5 min and different electrolyte amount (*i.e.,* 100 μL with one piece of the separator and 200 μL with two pieces of separators). Error bars represent t-test of performance from cycle to cycle at the same charging protocol. Notes: As shown in **Supplementary Fig. 32**, there is no significant difference in performance between the two cells. We used glass microfiber separators (GF/A, Whatman) with a diameter of 20 mm. Based on our findings, 100 μL of electrolyte is the recommended amount to fully infiltrate one piece of the separator. Further reducing the electrolyte amount for each separator could compromise the electrode-electrolyte wettability, which is crucial for optimal performance.

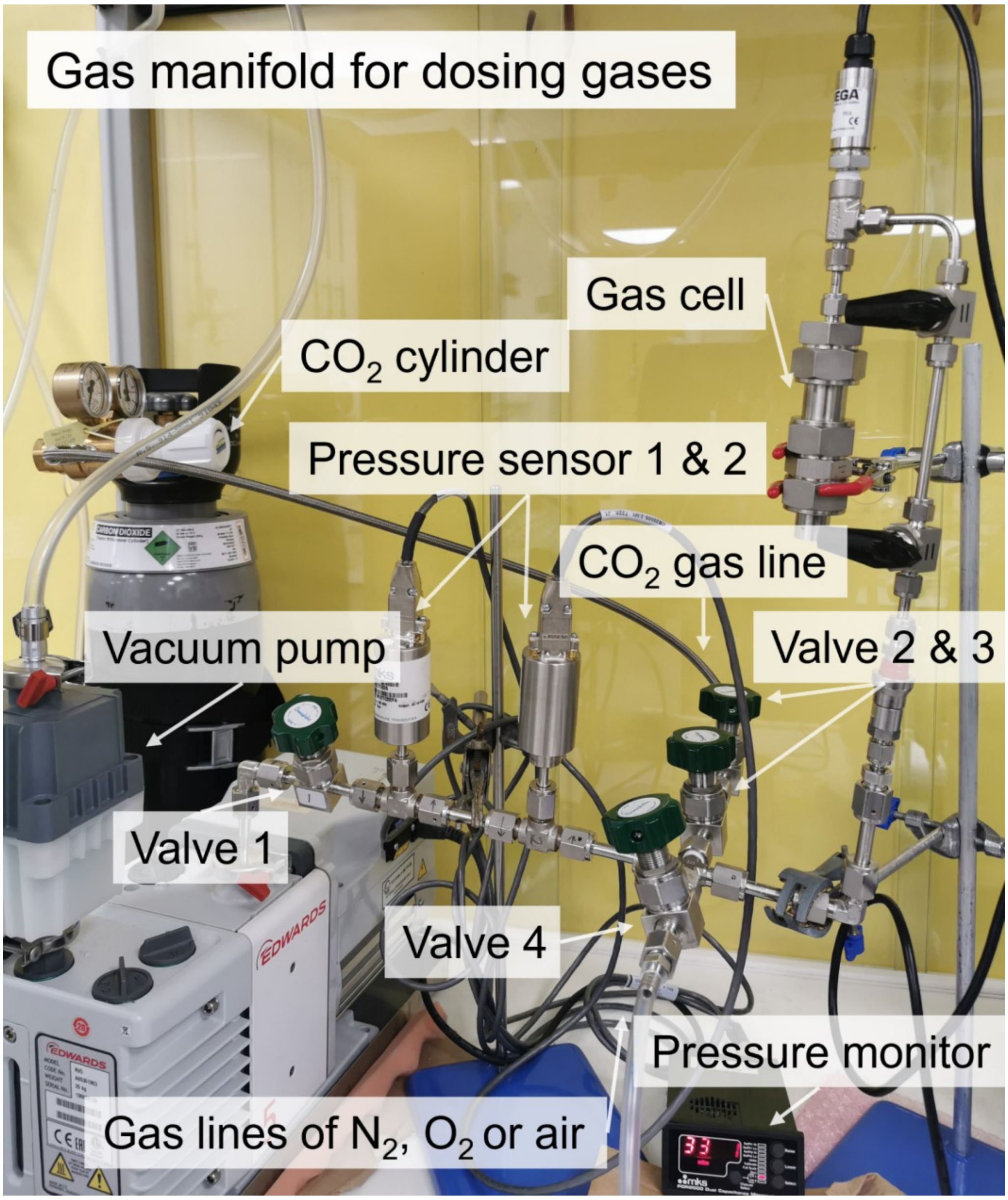

Supplementary Fig. 33. The setup of the gas manifold for dosing gases. A photo of the gas manifold for dosing gases into the electrochemical gas cell, where Valve 1 was used to control the connection between vacuum pump and gas cell, Valves 2 & 3 were used to control the connection between CO_2 cylinder and gas cell, and Valve 4 was used to control the connection between other gas cylinders and gas cell.

Supplementary Table 1. Pore structure details of carbon electrodes.

	S_{BET}^{a} (m^2 g^{-1})	V_t^{b} (cm^3 g^{-1})	D_A^{c} (nm)
**YP50F**	1694	0.73	0.87
**YP80F**	2324	1.14	1.13
**ACC-10**	1094	0.43	0.51
**ACC-20**	2004	0.82	0.88
**CMK-3**	353	1.02	3.71
**O-YP80F-1**	2216	1.08	1.19
**O-YP80F-7**	2243	1.09	1.18

Notes: [a]Brunauer–Emmett–Teller (BET) specific surface area; [b]Total pore volume; [c]average pore diameter.

Supplementary Table 2. Functional group details of carbon electrodes.

	**C (at%)**	**O (at%)**	**Si (at%)**
**YP50F**	93.51 ± 0.28	**6.49** ± 0.02	N/A
**YP80F**	94.53 ± 0.28	**5.14** ± 0.04	N/A
**ACC-10**	78.04 ± 0.35	13.96 ± 0.09 (**6.40**)	8.20 ± 0.03
**ACC-20**	86.43 ± 1.58	9.77 ± 0.15 (**6.34**)	3.80 ± 0.02
**CMK-3**	96.55 ± 0.21	**3.41** ± 0.07	N/A
**O-YP80F-1**	92.08 ± 0.22	**7.88** ± 0.05	N/A
**O-YP80F-7**	91.81 ± 0.19	**8.13** ± 0.01	N/A

Notes: Oxygen amount in the ( ) is the O-C amount, excluding the O-Si amount using the fitting of O1s spectra; SiO_2 was added as reinforcement particles in the commercial free-standing carbon electrodes, which is electrochemically inactive in the aqueous electrolyte. Error bars represent the standard deviation of atomic percentages of elements at different spots on the same sample. N/A (not available).

Supplementary Table 3. Performance comparison between various CO_2 capture technologies.

	CO_2 adsorption capacity (mmol kg^{-1})	CO_2 adsorption capacity (mmol L^{-1})	CO_2 adsorption rate (mmol kg^{-1} h^{-1})	Electrical Energy consumption (kJ mol^{-1})
**SSA-1[9] (15% CO_2, 85% N_2)**	273	40	301	179
**SSA-2[10] (15% CO_2, 85% N_2)**	485	133	223	311
**Redox-active AzPy[11] (15% CO_2, 5% O_2, 80% N_2)**	N/A	N/A	N/A	120
**Redox-active quinone-1[12] (15% CO_2, 5% O_2, 80% N_2)**	N/A	N/A	N/A	50-200
**Redox-active quinone-2[13] (15-100% CO_2, 85-0% N_2)**	0.8-1.3 $\mu mol_{CO2}/\mu mol_{quinone}$	N/A	N/A	40–90
**BPMED[14] (pure CO_2)**	N/A	N/A	N/A	148−325
**EMAR[15] (15% CO_2, 85% N_2)**	0.12-0.62 mol_{CO2}/mol_{amine}	N/A	N/A	40-120
**PCET-1[16,17] (15% CO_2, 85% N_2)**	N/A	N/A	N/A	60-145
**PCET-2[18] (20% CO_2, 3-20% O_2, 77-60% N_2)**	N/A	860-1410	N/A	36–55
**Amine-based separation (flue gas)**	N/A	N/A	N/A	~65[14]<br>~90[19]<br>~120[20]<br>~100[21]
**This work (pure CO_2)**	~170 (1.6 V)	~90	~350	~20
**This work (ca. 20% CO_2, 15% O_2, 65% N_2)**	~83 (0.8 V)	~44	~310	~20

Notes: SSA (Supercapacitive swing adsoprtion), AzPy (4,4′-azopyridine), BPMED (Bipolar membrane electrodialysis), EMAR (Electrochemically mediated amine regeneration), PCET (Proton-coupled electron transfer with derivatized phenazine), N/A (not available). The volumetric capacity is normalized by the volume of the gas-exposed working electrode.

Supplementary References

1 Xu, Z., Xie, F., Wang, J., Au, H., Tebyetekerwa, M., Guo, Z., Yang, S., Hu, Y. S. & Titirici, M. M. All-Cellulose-Based Quasi-Solid-State Sodium-Ion Hybrid Capacitors Enabled by Structural Hierarchy. *Adv. Funct. Mater.* **29**, 1903895, (2019).

2 Binford, T. B., Mapstone, G., Temprano, I. & Forse, A. C. Enhancing the capacity of supercapacitive swing adsorption CO2 capture by tuning charging protocols. *Nanoscale* **14**, 7980-7984, (2022).

3 Zhu, S., Li, J., Toth, A. & Landskron, K. Relationships between Electrolyte Concentration and the Supercapacitive Swing Adsorption of CO2. *ACS Appl. Mater. Interfaces* **11**, 21489-21495, (2019).

4 Zhu, S., Li, J., Toth, A. & Landskron, K. Relationships between the Elemental Composition of Electrolytes and the Supercapacitive Swing Adsorption of CO2. *ACS Appl. Energy Mater.* **2**, 7449-7456, (2019).

5 Gago, A. S., Ansar, S. A., Saruhan, B., Schulz, U., Lettenmeier, P., Cañas, N. A., Gazdzicki, P., Morawietz, T., Hiesgen, R., Arnold, J. & Friedrich, K. A. Protective coatings on stainless steel bipolar plates for proton exchange membrane (PEM) electrolysers. *J. Power Sources* **307**, 815-825, (2016).

6 Bredar, A. R. C., Chown, A. L., Burton, A. R. & Farnum, B. H. Electrochemical Impedance Spectroscopy of Metal Oxide Electrodes for Energy Applications. *ACS Appl. Energy Mater.* **3**, 66-98, (2020).

7 Krewer, U., Röder, F., Harinath, E., Braatz, R. D., Bedürftig, B. & Findeisen, R. Review—Dynamic Models of Li-Ion Batteries for Diagnosis and Operation: A Review and Perspective. *J. Electrochem. Soc.* **165**, A3656-A3673, (2018).

8 Liu, Y., Lucas, É., Sullivan, I., Li, X. & Xiang, C. Challenges and opportunities in continuous flow processes for electrochemically mediated carbon capture. *iScience* **25**, 105153, (2022).

9 Bilal, M., Li, J. & Landskron, K. Enhancing Supercapacitive Swing Adsorption of CO2 with Advanced Activated Carbon Electrodes. *Adv. Sustainable Syst.* **7**, 2300250, (2023).

10 Bilal, M., Li, J., Guo, H. & Landskron, K. High-Voltage Supercapacitive Swing Adsorption of Carbon Dioxide. *Small* **19**, 2207834, (2023).

11 Li, X., Zhao, X., Liu, Y., Hatton, T. A. & Liu, Y. Redox-tunable Lewis bases for electrochemical carbon dioxide capture. *Nat. Energy* **7**, 1065-1075, (2022).

12 Diederichsen, K. M., Liu, Y., Ozbek, N., Seo, H. & Hatton, T. A. Toward solvent-free continuous-flow electrochemically mediated carbon capture with high-concentration liquid quinone chemistry. *Joule* **6**, 221-239, (2022).

13 Voskian, S. & Hatton, T. A. Faradaic electro-swing reactive adsorption for CO2 capture. *Energy Environ. Sci.* **12**, 3530-3547, (2019).

14 Eisaman, M. D., Alvarado, L., Larner, D., Wang, P., Garg, B. & Littau, K. A. CO2 separation using bipolar membrane electrodialysis. *Energy Environ. Sci.* **4**, 1319-1328, (2011).

15 Wang, M., Herzog, H. J. & Hatton, T. A. CO2 Capture Using Electrochemically Mediated Amine Regeneration. *Ind. Eng. Chem. Res.* **59**, 7087-7096, (2020).

16 Jin, S., Wu, M., Gordon, R. G., Aziz, M. J. & Kwabi, D. G. pH swing cycle for CO2 capture electrochemically driven through proton-coupled electron transfer. *Energy Environ. Sci.* **13**, 3706-3722, (2020).

17 Jin, S., Wu, M., Jing, Y., Gordon, R. G. & Aziz, M. J. Low energy carbon capture via

electrochemically induced pH swing with electrochemical rebalancing. *Nat. Comm.* **13**, 2140, (2022).

18 Pang, S., Jin, S., Yang, F., Alberts, M., Li, L., Xi, D., Gordon, R. G., Wang, P., Aziz, M. J. & Ji, Y. A phenazine-based high-capacity and high-stability electrochemical CO2 capture cell with coupled electricity storage. *Nat. Energy* **8**, 1126-1136, (2023).

19 Hamdy, L. B., Goel, C., Rudd, J. A., Barron, A. R. & Andreoli, E. The application of amine-based materials for carbon capture and utilisation: an overarching view. *Mater. Adv.* **2**, 5843-5880, (2021).

20 Dutcher, B., Fan, M. & Russell, A. G. Amine-Based CO2 Capture Technology Development from the Beginning of 2013—A Review. *ACS Appl. Mater. Interfaces* **7**, 2137-2148, (2015).

21 Zito, A. M., Clarke, L. E., Barlow, J. M., Bím, D., Zhang, Z., Ripley, K. M., Li, C. J., Kummeth, A., Leonard, M. E., Alexandrova, A. N., Brushett, F. R. & Yang, J. Y. Electrochemical Carbon Dioxide Capture and Concentration. *Chem. Rev.* **123**, 8069-8098, (2023).
